# Ovarian localization drives IL-7R-dependent CD8 coreceptor expression in peripheral double-negative T cells

**DOI:** 10.1007/s00018-026-06295-x

**Published:** 2026-06-16

**Authors:** Toni Martin, Howard A. Young, Enitome E. Bafor

**Affiliations:** 1 Cancer Innovation Laboratory, Center for Cancer Research, National Cancer Institute, Frederick, MD 21702 USA; 2 Department of Infectious Diseases and Immunology, College of Veterinary Medicine, University of Florida, Gainesville, Fl 32608 USA

**Keywords:** T cell plasticity, Reproductive immunology, Tissue conditioning, Lymphocyte adaptation, Immune homeostasis, Ovarian immunity

## Abstract

The ovary is an immunologically dynamic tissue that coordinates recurrent inflammation-like remodeling while preserving local T cell tolerance, yet whether it actively instructs infiltrating lymphocyte phenotype or passively enriches pre-existing subsets has not been directly tested. To address this, we used peripheral double-negative T cells (DNTs; CD3^+^CD4^−^CD8^−^NK1.1^−^) as a coreceptor-null system to detect tissue-imposed phenotypic change after ovarian entry. Using adoptive transfer, labeled splenic DNTs accumulated in the ovary relative to other tissue sites, and donor DNTs acquired surface CD8 expression within 4 days of ovarian localization. Dissociated ovarian cells were sufficient to drive CD8 upregulation on sorted DNTs in vitro, supporting that ovarian cellular cues can promote this response. Ovarian single-cell RNA-seq provided supportive evidence for *Il7*-expressing stromal subsets, and an *Il7r*-enriched DNT-associated lymphoid population, findings compatible with local IL-7-associated microenvironmental cues that may contribute to conditioning infiltrating T cells. Functionally, IL-7R was required for efficient CD8 upregulation after ovarian localization, and IL-7R or IL-7 deficiency shifted the endogenous ovarian DNT: CD8^+^ T cell balance. These findings support the ovary as an immune-conditioning environment in which tissue entry is associated with CD8 coreceptor induction on peripheral DNTs, and identify IL-7R signaling as a requirement for efficient induction in this setting.

**Supplementary Information:**

The online version contains supplementary material available at 10.1007/s00018-026-06295-x.

## Introduction

The ovary is an immunologically active organ that sustains recurrent cycles of follicle growth, ovulation, corpus luteum formation, and regression, each of which requires coordinated leukocyte participation and precise tissue remodeling [1–4]. Ovulation is a physiologic inflammatory event characterized by proteolysis, vascular changes, and immune cell recruitment, yet these processes must remain tightly controlled to preserve follicular integrity, endocrine function, and fertility [1, 2]. T cells are among the immune populations recruited to the ovary during these cycles and have been identified in both murine and human ovarian tissue under homeostatic conditions [5–7]. Their participation in normal ovarian physiology raises a fundamental question on whether the ovary actively instructs the phenotype of infiltrating T cells, or whether it simply enriches pre-existing subsets through recruitment and retention. Resolving which of these mechanisms operates has direct implications for interpreting how disruptions to this balance contribute to reproductive dysfunction conditions such as premature ovarian insufficiency, autoimmune ovarian disease, infertility, and ovarian tumor immune surveillance. Thus, this question is central to understanding how ovarian immune balance is maintained across recurrent cycles of physiologic remodeling [5, 6, 8, 9].

Across multiple non-lymphoid organs, tissue microenvironments have been shown to exert stable and reversible phenotypic changes on infiltrating lymphocytes after tissue entry, through integrated cytokine, chemokine, adhesion, and antigen-presenting signals [10, 11]. Such tissue-conditioning capacity is well documented in barrier and mucosal sites, where local signals drive the acquisition of residency-associated surface markers, transcriptional adaptations, and altered functional states in T cells that differ from their circulating counterparts [10, 11]. Importantly, the nature and extent of phenotypic adaptation vary by tissue, reflecting organ-specific microenvironment [10]. Whether the ovary, a non-barrier reproductive organ undergoing cyclic remodeling, similarly encodes tissue-conditioning capacity for infiltrating peripheral T cells has not been directly tested.

Surface expression of CD4 and CD8 coreceptors amplifies TCR signaling by stabilizing peptide-MHC interactions and recruiting Lck to the TCR complex, thereby lowering activation thresholds and shaping the quality of the downstream T cell response [12–14]. Coreceptor expression is thus functionally consequential with gain or loss of CD8 at the surface level alters the sensitivity of a T cell to MHC class I-restricted antigens and changes its integration of homeostatic signals, including those transmitted through IL-7R [15, 16]. Though coreceptor expression is canonically fixed following thymic lineage commitment, this state is not stably maintained in the periphery. Cytokine signals and tissue-derived cues can modulate CD8 expression on mature T cells under defined conditions [17, 18], and the functional consequences of acquiring or losing coreceptor surface expression extend beyond phenotype classification to include altered antigen sensitivity and homeostatic cytokine responsiveness. Whether such tissue-imposed coreceptor modulation occurs in the ovary, and which local signals mediate it, remains unclear. In addition, among peripheral DNTs, CD8 is the coreceptor more consistently reported to be re-expressed in response to cytokine and activation signals, making it the biologically relevant transition to track in this population [17–20].

Peripheral DNTs (CD3^+^CD4^−^CD8^−^NK1.1^−^) constitute approximately 1–5% of T lymphocytes in peripheral blood and lymphoid organs of mice and humans [8, 9, 21]. These cells have been identified in the female reproductive tract at notably higher proportions relative to other tissues, where they have been attributed regulatory functions relevant to local immune tolerance [21–23]. DNTs have also been characterized in human genital tissues as a transcriptionally distinct, functionally heterogeneous population with tissue-resident features [24]. In addition, their coreceptor-null state is not a terminally fixed fate; cytokine context and local signals can influence coreceptor re-expression, including CD8 upregulation under defined conditions [17, 18]. This plasticity, combined with the baseline absence of CD4 and CD8, positions DNTs as a tractable system for detecting de novo tissue-imposed coreceptor modulation, where any CD8 signal acquired after ovarian localization cannot be attributed to pre-existing coreceptor expression. Thus, providing a direct readout of ovarian immune-conditioning capacity.

Here, we tested the model that the ovary acts as an immune-conditioning site capable of modulating peripheral T cell coreceptor state after tissue entry. Using adoptive transfer of peripheral DNTs, we asked whether ovarian localization preferentially supports their accumulation and whether tissue entry is followed by CD8 acquisition. We then used an in vitro ovarian coculture system to determine whether ovarian cells are sufficient to promote this response. To define candidate local signals underlying this remodeling, we analyzed a published ovarian single-cell RNA-seq dataset to map stromal and endothelial features consistent with candidate immune conditioning microenvironments. We used targeted pathway perturbation to test which T cell-intrinsic signals are required for efficient CD8 upregulation after ovarian localization and for maintenance of endogenous ovarian DNT: CD8^+^ T cell balance. Using DNTs as a coreceptor-null experimental system, this study identifies a tissue-level association by which ovarian localization is linked to phenotypic changes in infiltrating T cells and provides a new framework for interpreting ovarian immune phenotypes in homeostasis and tolerance-relevant dysfunction.

## Materials and methods

### Animals

All experiments used female C57BL/6J mice aged 3–4 months. Strains included wild-type (WT; JAX stock #000664), IFN-γ^−/−^ (JAX stock #002287; [25]), IFN-γR^−/−^ (JAX stock #003288 [26]), WT GFP^+^ mice (JAX stock # 006567 [27]), IL7R^−/−^ (JAX stock #002295 [28]) and IL-7^−/−^ (DNAX Research Institute of Cellular and Molecular Biology, CA [29]) and mice heterozygous for the AU-rich element replacement in *Ifng* (ARE^+/−^ ). Generation of ARE mice has been previously described [30, 31]. Briefly, a 162-nucleotide AU-rich element sequence within the 3′ untranslated region of *Ifng* was deleted by *Cre/lox*-mediated recombination and replaced with a neutral sequence via electroporation of embryonic stem cells. Chimeric mice derived from the modified locus were backcrossed for more than ten generations onto the C57BL/6 background [30, 31]. Mice were housed and bred at the National Cancer Institute at Frederick animal facility under a 12 h light-dark cycle with *ad libitum* access to food and water. Estrous stage was assessed by vaginal lavage on the day of experimentation as described below. Mice were euthanized by isoflurane overdose, and ovaries, uteri, spleens, and reproductive tissue-draining lymph nodes (dLNs; medial iliac) were harvested for downstream assays. All procedures complied with the NIH Guide for the Care and Use of Laboratory Animals and are reported in accordance with the ARRIVE guidelines. Protocols were approved by the NCI Animal Care and Use Committee (Frederick, MD; protocol #21 − 018).

### Estrous cycle staging

Estrous cycle stage was determined by vaginal lavage as described previously [32, 33]. Briefly, 50–100 µL of lavage fluid was applied to clean glass microscope slides (Corning), air-dried at room temperature, fixed in cold 100% methanol, and stained with 0.01% methylene blue (Sigma-Aldrich) [33]. Stained smears were examined by light microscopy and assigned to estrous stage based on standard cytological criteria. To synchronize cycles before experimentation, females were co-housed with male-soiled bedding for 48 h [34]. Vaginal lavage samples were collected at the end of each experiment to confirm estrous stage.

### Tissue processing and single-cell preparation

Tissues were harvested on the day of experimentation and placed immediately into cold RPMI 1640 (Corning) supplemented with 10% fetal bovine serum (FBS) for lymphoid tissues or 20% FBS for reproductive tissues, 100 U/mL penicillin, 100 µg/mL streptomycin, and 2 mM L-glutamine.

#### Reproductive tissues (ovary and uterus)

Ovaries and uteri were trimmed of adipose and connective tissue and transferred into digestion buffer consisting of RPMI 1640 supplemented with collagenase type IV (0.01 mg/mL; Sigma-Aldrich) and, where required, DNase I (4 U/mL; Sigma-Aldrich) to reduce cell aggregation. Total digestion volume was adjusted to 5 mL with supplemented RPMI. Tissues were minced into small fragments and enzymatically dissociated using a gentleMACS Octo Dissociator with heaters (Miltenyi Biotec) under a custom program (37 °C, 20 min, 400 rpm) [33]. Digests were immediately transferred to ice and diluted with cold RPMI to halt enzymatic activity. Cell suspensions were passed through a 40 μm strainer, centrifuged (1,200 x g, 5 min, 4 °C), and washed. Where erythrocyte contamination was present, red blood cell lysis was performed using ACK lysis buffer (Quality Biological) for 5 min at room temperature, quenched with excess PBS, and washed.

#### Lymphoid tissues (spleen and dLNs)

Spleens and dLNs were dissociated by gentle mechanical disruption in Filtra blender bags (Labplas) as described previously [33]. Each spleen or pooled dLN set was placed in a Filtra pouch with approximately 3 mL RPMI, gently mashed, and rinsed with 10 mL PBS. Cell suspensions were collected from the filter side of the pouch into 50 mL tubes and centrifuged (1,200 x g, 5 min, 4 °C). Pellets were resuspended in 5 mL ACK lysis buffer for 5 min at room temperature, quenched with 20 mL PBS, washed, resuspended in cold FACS buffer (PBS containing 2% FBS and 2 mM EDTA), and passed through a 40 μm strainer.

Final cell pellets from all tissues were resuspended in cold FACS buffer and enumerated prior to antibody staining (Table S1) or downstream assays [33]. Leukocyte counts were determined using a Sysmex hematology analyzer, and total cell counts were measured using a Cellometer Auto T4 (Nexcelom Bioscience).

### Adoptive transfer of DNTs

Splenic DNTs were FACS-sorted from female WT C57BL/6J donors using the gating strategy described below (live CD3^+^CD4^−^CD8α^−^NK1.1^−^). Sorted cells were washed and resuspended in sterile PBS at 1.5 × 10^5^ cells per 100 µL (Because splenic DNTs are a relatively rare population and sort yield varies between experiments, adoptive transfers were standardized to 1.5 × 10^5^ donor DNTs per mouse). Recipient females were cycle-synchronized by exposure to male-soiled bedding for 48 h prior to transfer. Cells were administered by intraperitoneal (i.p.) injection. Recipient mice were euthanized at 4 or 14 days post-transfer, as indicated for each experiment, and ovaries, uteri, spleens, and dLNs were harvested for flow cytometric analysis.

### Cell labelling experiments

In experiments requiring donor cell tracking, FACS-sorted splenic DNTs were labeled prior to transfer with either Qtracker 525 (QT; Invitrogen) or used as GFP^+^ cells sorted from ubiquitous GFP^+^ donors. Qtracker 525 labeling was performed according to the manufacturer’s instructions; this reagent comprises CdSe/ZnS core–shell quantum dot nanoparticles (~ 10–15 nm) coupled to a delivery peptide that mediates cytoplasmic vesicular uptake [35, 36]. QT-labeled or GFP⁺ DNTs (1.5 × 10^5^ per mouse) were transferred i.p. into WT, ARE^+/−^, IFNγ^−/−^, or IFNγR^−/−^ recipients as described above. Qtracker 525 and GFP were used for donor-cell identification after transfer and were not used as proliferation-tracking reagents in this study.

### In vitro coculture assays

To test whether ovarian cells are sufficient to induce CD8 expression on DNTs, FACS-sorted splenic DNTs were labeled with CellTrace Yellow (Thermo Fisher Scientific) per manufacturer instructions and co-cultured with enzymatically dissociated total ovary cells in flat-bottom 96-well plates. Each well contained 2.5 × 10^4^ DNTs and 5 × 10^3^ ovary cells in complete medium consisting of RPMI 1640 (Thermo Fisher Scientific) supplemented with 10% FBS (VWR), 1 mM sodium pyruvate, 10 mM HEPES, non-essential amino acids, 2 mM L-glutamine (Sigma-Aldrich), 50 µM 2-mercaptoethanol, 100 U/mL penicillin, and 100 µg/mL streptomycin (Thermo Fisher Scientific). Cultures were established in technical duplicates with or without anti-CD3/CD28 Dynabeads (Thermo Fisher Scientific; 2 µL per well) and incubated for 72 h at 37 °C in a humidified incubator with 5% CO_2_. Cells were harvested at the end of culture for flow cytometric analysis.

### Flow cytometry and cell sorting

Single-cell suspensions (typically 2–5 × 10^6^ cells per sample) were prepared in 5 mL polystyrene tubes. Viability was assessed using Zombie Aqua fixable viability dye (BioLegend) at 1:50 dilution for 30 min at room temperature in the dark, followed by washing in FACS buffer. Fc receptors were blocked with anti-CD16/CD32 (clone 2.4G2; BD Biosciences) diluted 1:10 in 20 µL FACS buffer for 10 min at 4 °C. Fluorochrome-conjugated antibodies (Table S1) were added to the Fc block suspension and incubated for 30 min at 4 °C in the dark. Cells were washed, centrifuged (400 x g, 5 min, 4 °C), and resuspended in FACS buffer for acquisition. Compensation was performed using single-stained compensation beads and single-stained cell controls. FMO controls were used to set gates for all markers. Data were acquired on a BD LSRFortessa, BD FACSymphony A5, or BD FACSymphony S6 (BD Biosciences) using FACSDiva software and analyzed with FlowJo v10.10.0 (BD Biosciences). For CD4 versus CD8 display plots, representative panels were formatted using a consistent quadrant-based gating scheme within each experiment to improve comparability across figures.

For analysis, cell debris and residual erythrocytes were excluded by FSC-A versus SSC-A gating, and doublets were removed by FSC-A versus FSC-H gating. In adoptive transfer experiments, donor cells were identified as Qdot^+^ or GFP^+^ events within the live gate prior to downstream subset definition. CD8^+^ T cells were defined as CD3^+^CD8α^+^CD4^−^. DNTs were defined as live CD3^+^CD4^−^CD8α^−^NK1.1^−^. Within the CD8^+^ gate, effector/effector-memory cells were defined as CD44^hi^CD62L^lo^ and naive cells as CD44^lo^CD62L^hi^. Representative full gating strategies for the major experimental workflows are shown in Supplementary Fig. S1-3; for adoptive-transfer experiments, the same gating hierarchy was applied across ovary, uterus, spleen, and dLNs.

For cell sorting, splenic DNTs were purified on a BD FACSymphony S6 sorter using the gating strategy described above and collected into RPMI with 10% FBS. Post-sort purity was confirmed by immediate re-analysis and was routinely greater than 90%.

### Single cell RNA-seq analysis

Publicly available ovarian single cell RNA-seq data were accessed from GEO accession GSE232309 [37]. The original dataset comprised ovaries from young (3 month old; *n* = 4) and reproductively aged (9-month-old; *n* = 4) wild-type C57BL/6J mice, as reported by the source study. For the present re-analysis, only young ovary samples were used. The immune compartment comprised 760 cells from young ovary samples across 4 mice (range: 125–263 cells per mouse) and 1,269 cells from aged ovary samples across 4 mice, as retained after published QC filtering. For T-lineage analysis, 99 cells from young ovary samples fell within the lymphoid region. Processed Seurat objects were downloaded and imported into R (v4.5.2; RStudio 2026.01.0 + 392) using the Seurat and SeuratObject packages [38–42]. Objects corresponding to immune cells, stromal cells, granulosa cells, and endothelial/epithelial compartments were loaded from archived .rds files (GSE232309_Immune_Cells.rds.gz, GSE232309_Stroma_Cells.rds.gz, GSE232309_Granulosa_Cells.rds.gz, GSE232309_Endothelial_Epithelial_Cells.rds.gz), decompressed as required. All analyses used the RNA assay (DefaultAssay = “RNA”). Metadata were inspected to confirm cell numbers and sample representation, and published dataset annotations (cluster.names and/or celltype_annot) were retained for population labeling and summary visualization. Population clustering was not recomputed de novo from raw counts for the purposes of this manuscript; instead, the published processed Seurat objects and their corresponding dataset annotations were used as the basis for focused re-analysis and summary visualization.

Analyses focused on three components relevant to ovarian IL-7 axis biology. Within the immune compartment, a lymphoid-enriched subset was generated by selecting T and innate lymphoid populations from dataset annotations, followed by marker-guided classification of T-lineage states. A conservative transcript-based DNT definition was applied, identifying cells with low or absent *Cd4* and *Cd8a/Cd8b1* expression and minimal NK-associated signal (low *Nkg7*, *Gzmb*, and *Prf1*); CD4^+^ and CD8^+^ T cell classes were defined using conventional marker patterns. Because this analysis was based on transcript detection, and because sparse gene detection is inherent to scRNA-seq, all DNT-related classifications were interpreted conservatively as transcript-defined populations; all other T-lineage annotations were retained from the published dataset. These transcript-defined classes are therefore used as descriptive analytical categories and are not intended to imply protein-level equivalence to the flow cytometric definitions used elsewhere in the manuscript. Furthermore, since scRNA-seq transcript dropout and ambient RNA contamination can affect sparse markers, DNT classification was applied conservatively using multiple co-expressed markers and reducing the likelihood that dropouts alone drive misclassification. Within the stromal compartment, *Il7* expression was quantified across stromal subtypes. Within granulosa and endothelial/epithelial compartments, expression of chemokines, adhesion molecules, and immune interaction genes (*Il7*, *Il7r*, *Cxcl12*, *Icam1*, *Vcam1*) was assessed to identify candidate routes for lymphocyte entry and tissue localization.

### Statistical analysis

Statistical analyses were performed in GraphPad Prism v10.6.1 (GraphPad Software) and R v4.5.2. Data are presented as mean ± SEM unless otherwise stated. Two-group comparisons used Mann-Whitney tests for non-parametric data or Welch’s t-tests when variances were unequal. Multi-group comparisons used Kruskal-Wallis tests for non-parametric data or one-way ANOVA with Tukey post hoc correction as appropriate. Correlations were assessed by Pearson’s correlation. A p-value below 0.05 was considered statistically significant. Statistical tests and biological replicate numbers (n) are reported in each figure legend.

## Results

### Peripheral DNTs preferentially accumulate in the ovary and upregulate surface CD8 expression after tissue entry

**Fig. 1:**
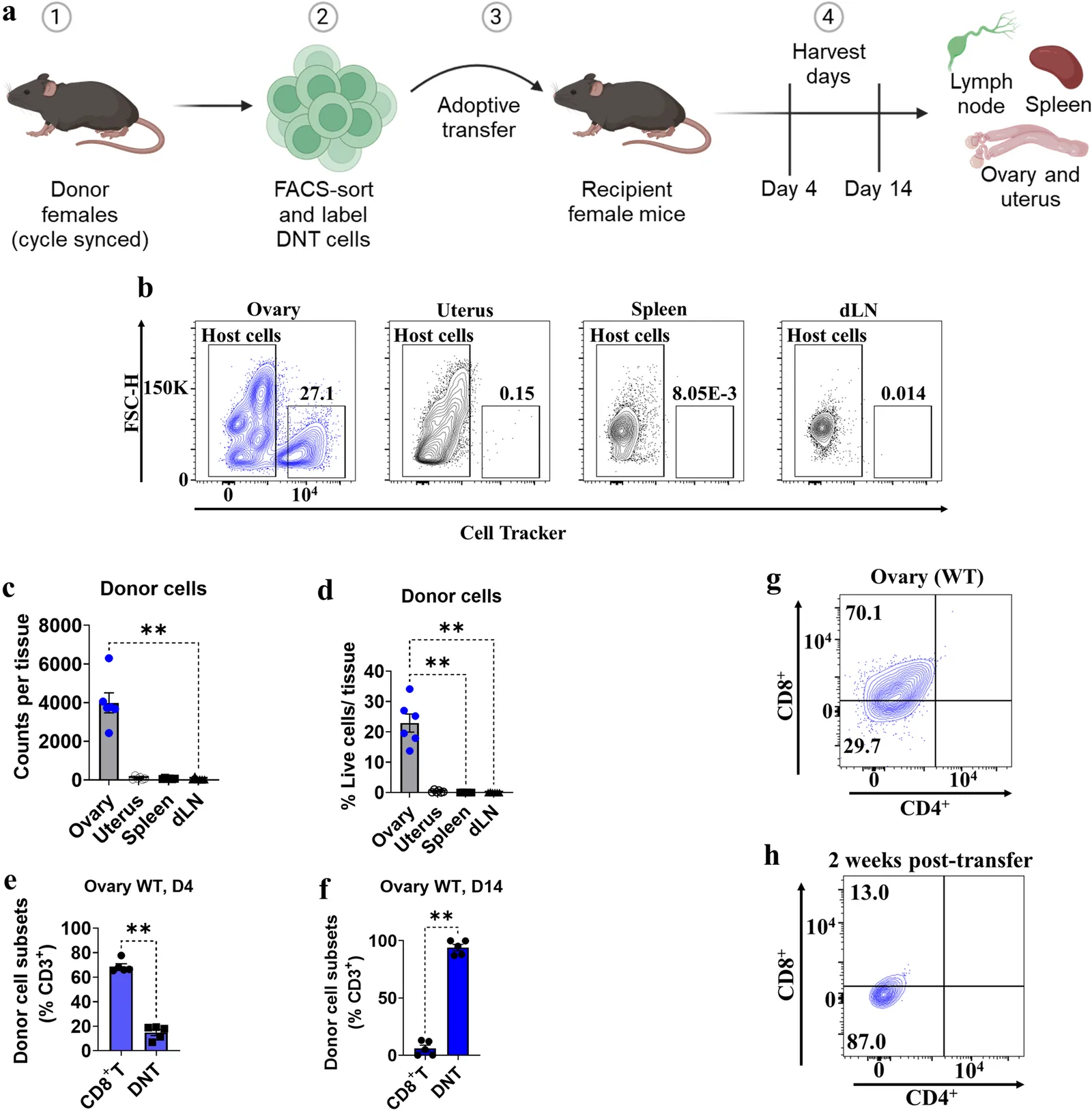
Peripheral DNTs are preferentially recovered from the ovary and upregulate surface CD8 expression after tissue entry.**a** Schematic of the adoptive transfer design, indicating tissues harvested and collection time points. **b** Representative flow cytometry plots showing labeled donor cells detected in the ovary, uterus, spleen, and reproductive tissue-draining lymph nodes (dLNs; medial and iliac) at day 4 post-transfer; Donor-cell labels were used specifically for tracking and identification after transfer and were not interpreted as proliferation readouts. **c**, **d** Donor cell numbers per tissue (**c**) and donor frequency among live cells (**d**) at day 4 (*n* = 6 mice; Kruskal-Wallis test). **e**, **f** Proportion of ovarian donor CD3^+^ T cells gated as CD8^+^ versus CD4^−^CD8^−^ at day 4 (**e**) and day 14 (**f**) post-transfer (*n* = 5 mice per time points; Mann-Whitney test). **g**, **h** Representative CD4 versus CD8 quadrant plots of ovarian donor CD3^+^ T cells at day 4 (**g**) and day 14 (**h**), corresponding to the quantified data in panels (**e**) and (**f**). Bars show mean ± SEM. **P < 0.01

To determine whether the ovary accumulates peripheral DNTs and alters their surface phenotype after tissue entry, FACS-sorted splenic DNTs were labeled and adoptively transferred into cycle-synchronized WT recipients, and donor cells were tracked across the ovary, uterus, spleen, and reproductive tissue-draining lymph nodes (dLNs) at day 4 post-transfer (Fig. 1a). Donor cells were consistently detected in the ovary but were rare in the uterus, spleen, and dLNs, with significantly higher numbers and live cell frequency in the ovary than in all other tissues examined (Fig. 1b-d), indicating that the ovarian microenvironment preferentially supports donor DNT accumulation.

To determine whether ovarian localization was accompanied by phenotypic change, donor cell coreceptor status was assessed after tissue entry. The transferred population was confirmed to be CD3^+^NK1.1^−^CD4^−^CD8α^−^ immediately before transfer, establishing a coreceptor-null baseline (Fig. S4a). At day 4, the majority of ovarian donor cells had expressed surface CD8, with only a minor fraction remaining CD4^−^CD8^−^ and by day 14, donor cells in the ovary had returned to a predominantly CD4^−^CD8^−^ phenotype, with minimal residual CD8 signal (Fig. 1e-h). Together, these data establish preferential ovarian accumulation of transferred DNTs and show that ovarian entry is associated with early, transient CD8 induction. Though the present design does not track the fate of each donor cell individually, the combination of pre-transfer donor control and the dynamic donor phenotype observed by day 14 supports interpretation of this pattern as transient CD8 acquisition and not a persistence of a stable pre-existing CD8-positive donor subset. In addition, since donor DNTs were administered intraperitoneally, we interpret this distribution as preferential ovarian recovery after i.p. transfer.

To determine whether ovarian cellular material was sufficient to promote CD8 induction under ex vivo conditions, sorted WT splenic DNTs were co-cultured with dissociated total ovary cells for 72 h in the presence or absence of CD3/CD28 costimulation (Fig. S4b). CD8 upregulation was detectable under both conditions (Fig. S4c-d), supporting that ovarian cellular material can promote CD8 induction on DNTs ex vivo. Because this assay used total dissociated ovarian cells, it does not identify the responsible ovarian cell population or mediator,

### IFN-γ signaling environment modulates CD8 upregulation on ovarian donor DNTs in a context-dependent manner

**Fig. 2:**
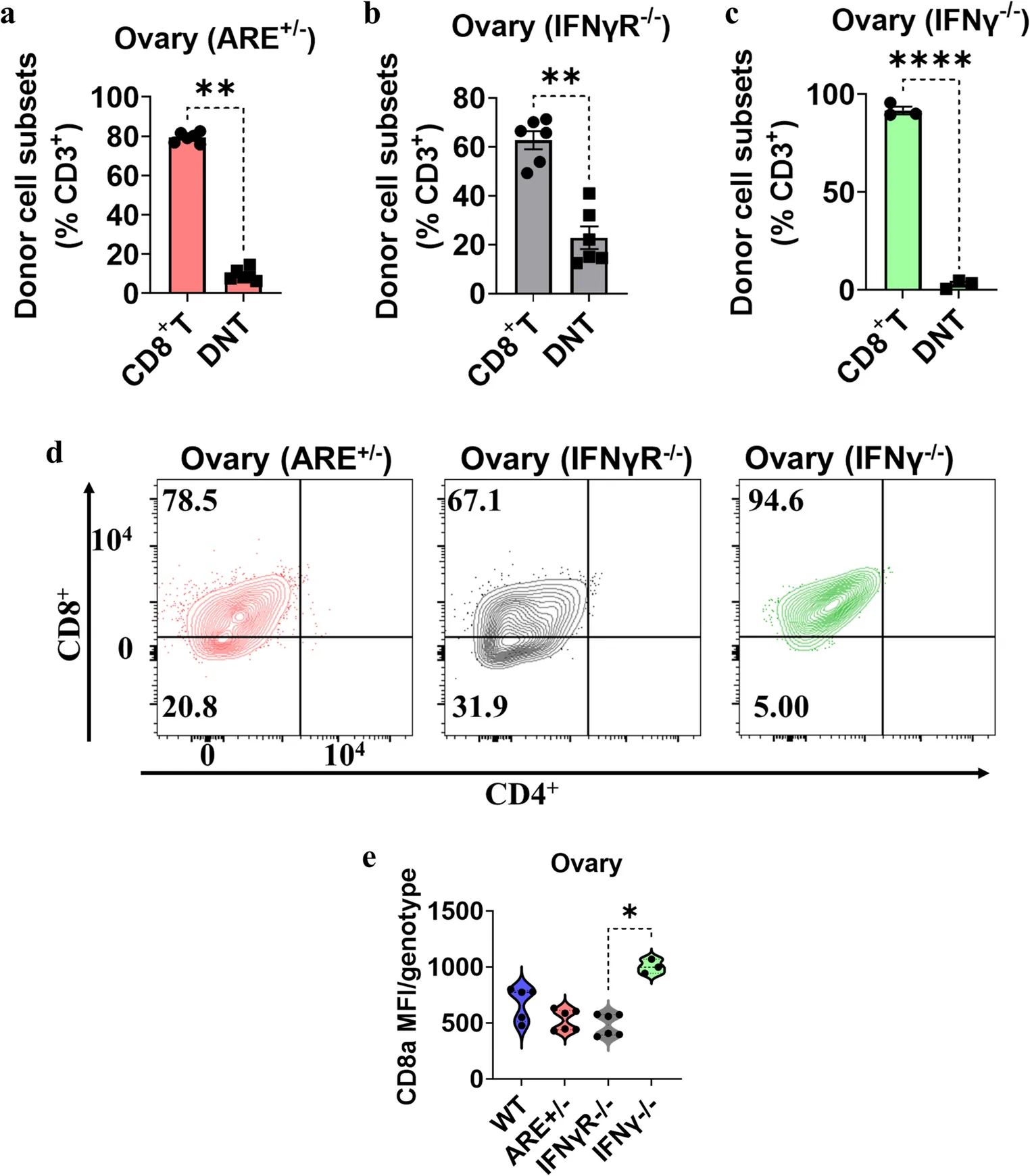
IFN-γ signaling in ovarian environment modulates the proportion and surface expression of CD8 upregulation on ovarian donor DNTs. **a**–**c** Proportion of ovarian donor CD3^+^ T cells that are CD8^+^ versus CD4^−^CD8^−^ in ARE^+/−^ mice (**a**), IFN-γR^−/−^ (**b**), and IFN-γ^−/−^ (**c**) recipients at day 4 post-transfer of sorted splenic DNTs (*n* = 3–6 mice per group; Mann-Whitney test and Welch’s t-test). **d** Representative CD4 versus CD8 quadrant plots of ovarian donor CD3^+^ T cells from each recipient genotype at day 4; numbers indicate the percentage of events within indicated gates. **e** CD8α MFI on ovarian donor CD3^+^ T cells across recipient genotypes (WT, ARE^+/−^, IFN-γR^−/−^, IFN-γ^−/−^), showing genotype-dependent differences in CD8 surface level (*n* = 3–6 mice per group; Kruskal-Wallis test). Bars show mean ± SEM in A-C; violin plots show individual values in E. *P < 0.05, **P < 0.01, ****P < 0.0001

Having established that ovarian localization drives CD8 upregulation, we asked whether this response is uniform across immune signaling contexts or varies depending on the recipient environment. Donor DNT phenotypes in ovaries at day 4 were compared across recipients with altered IFN-γ pathway function. In ARE^+/−^ recipients, the majority of ovarian donor cells were CD8^+^, with only a minor persistent DNT fraction (Fig. 2a). IFN-γR^−/−^ recipients showed a similar predominance of donor CD8^+^ cells, though a comparatively larger DNT fraction persisted (Fig. 2b). IFN-γ^−/−^ recipients showed near-complete donor CD8 upregulation, with very few cells remaining CD4^−^CD8^−^ (Fig. 2c-d). CD8α MFI on ovarian donor cells also differed across genotypes, with the highest signal observed in IFN-γ^−/−^ hosts (Fig. 2e). Thus, ovarian localization is associated with CD8 upregulation on transferred DNTs across recipient contexts, while the magnitude of this response is modulated by the surrounding IFN-γ signaling environment.

### Donor DNT-derived CD8^**+**^ cells in the ovary are phenotypically distinct from endogenous host ovarian CD8^**+**^ T cells

**Fig. 3:**
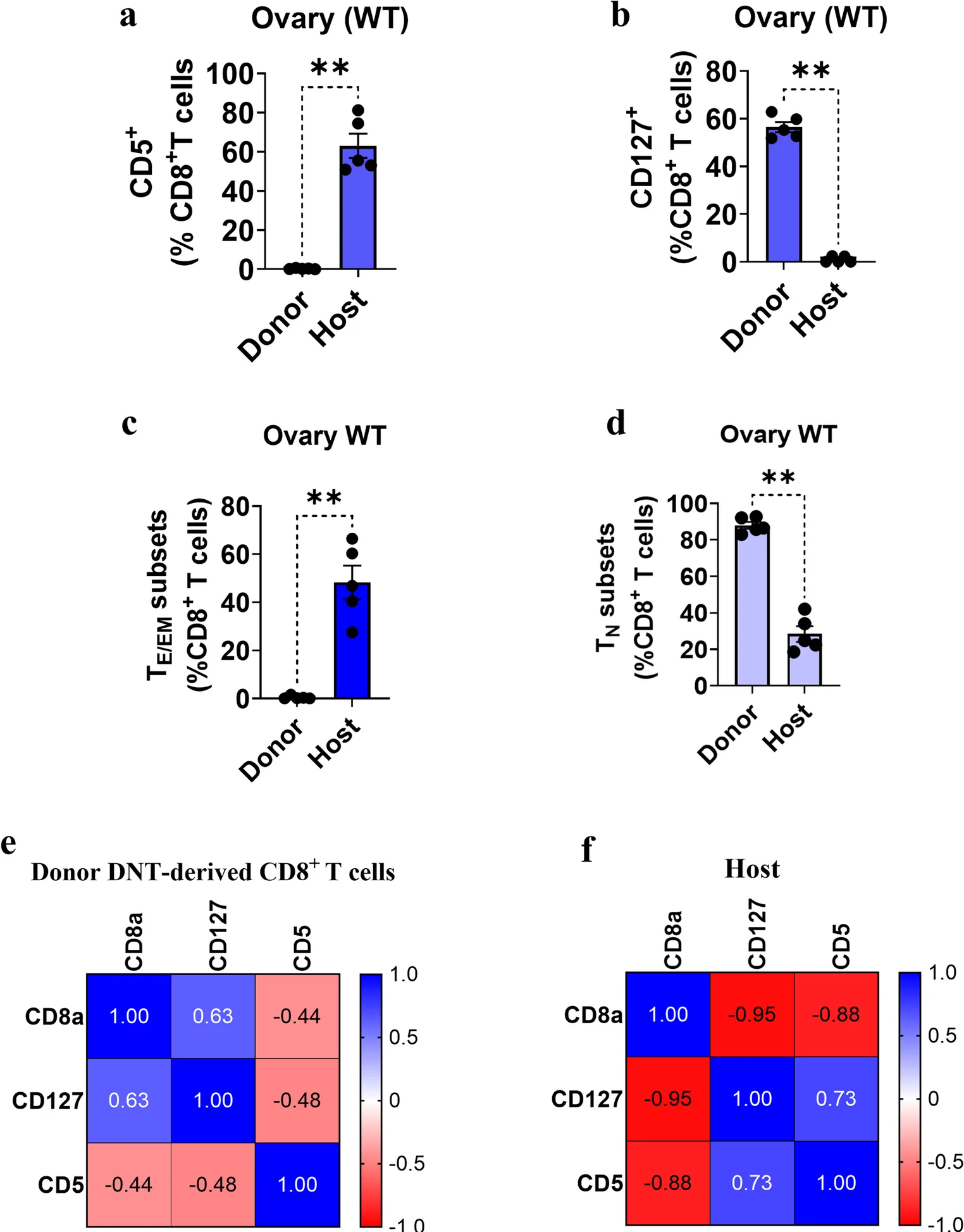
Donor DNT-derived CD8^+^cells in the ovary are phenotypically distinct from endogenous host ovarian CD8^+^T cells.**a**, **b** CD5 (**a**) and CD127/IL-7R (**b**) expression on donor DNT-derived CD8^+^ cells versus host ovarian CD8^+^ T cells at day 4 post-transfer (*n* = 5 mice; Mann-Whitney test). **c**, **d** Proportion of effector/effector-memory (T_E/EM_; CD44^hi^CD62L^lo^) (**c**), and naive (T_N_; CD44^lo^CD62L^hi^) (**d**) subsets among donor-derived and host ovarian CD8^+^T cells (*n* = 5 mice; Mann-Whitney test). **e**, **f** Pearson correlation matrices of CD8α, CD127 (IL-7R), and CD5 MFIs within the donor DNT-derived CD8^+^ gate (**e**) and the host ovarian CD8^+^ gate (**f**), showing opposing correlation patterns between the two compartments. Bars show mean ± SEM. **P < 0.01

Because ovarian localization drives CD8 upregulation on transferred DNTs, we asked whether the resulting donor-derived CD8^+^ cells resemble endogenous host ovarian CD8^+^ T cells or represent a distinct tissue-conditioned phenotype. At day 4 post-transfer in WT recipients, donor-derived CD8^+^ cells and host ovarian CD8^+^ T cells differed across multiple surface markers. Donor-derived cells were predominantly CD5^lo^, whereas host CD8^+^ T cells were largely CD5^+^ (Fig. 3a, Fig. S5a). Conversely, donor-derived cells showed strong CD127/IL-7R expression, while host CD8^+^ T cells showed minimal IL-7R expression at the same time point (Fig. 3b, Fig. S5b). With respect to differentiation status, donor-derived CD8^+^ cells were enriched for a CD62L^hi^CD44^lo^ naive-like phenotype, whereas host ovarian CD8^+^ T cells showed a substantial effector/effector-memory (T_E/EM_) population (Fig. 3c, Fig. S5c). Since the transferred donor population originated from spleen, this relative TN enrichment is not interpreted on its own as evidence of ovarian instruction but as supportive, together with the CD5^lo^ and IL-7R^hi^ phenotype, that donor-derived CD8^+^ cells remain phenotypically distinct from endogenous host ovarian CD8^+^ T cells after ovarian entry.

To determine whether these marker differences were coordinated within cells, Pearson correlations among CD8α, IL-7R, and CD5 MFIs were examined separately in each compartment. In donor-derived CD8^+^ cells, CD8α correlated positively with IL-7R (*r* = 0.63) and inversely with CD5 (*r* = -0.44), while IL-7R and CD5 were also inversely related (*r* = -0.48) (Fig. 3d). In host ovarian CD8^+^ T cells, the pattern was reversed: CD8α correlated inversely with both IL-7R (*r* = -0.95) and CD5 (*r* = -0.88), while IL-7R and CD5 were positively related (*r* = 0.73) (Fig. 3e). These opposing correlation structures indicate that CD8α, IL-7R, and CD5 are coupled differently in donor-derived cells than in host ovarian CD8^+^ T cells, reflecting different phenotypic co-regulation in each compartment. Collectively, these data demonstrate that ovarian CD8 induction in donor DNTs yields a phenotypically distinct CD8-expressing population.

### Ovarian scRNA-seq provides supportive evidence for *Il7*-expressing stromal subsets, a chemokine and adhesion landscape, and an *Il7r*-enriched DNT-associated lymphoid compartment

**Fig. 4:**
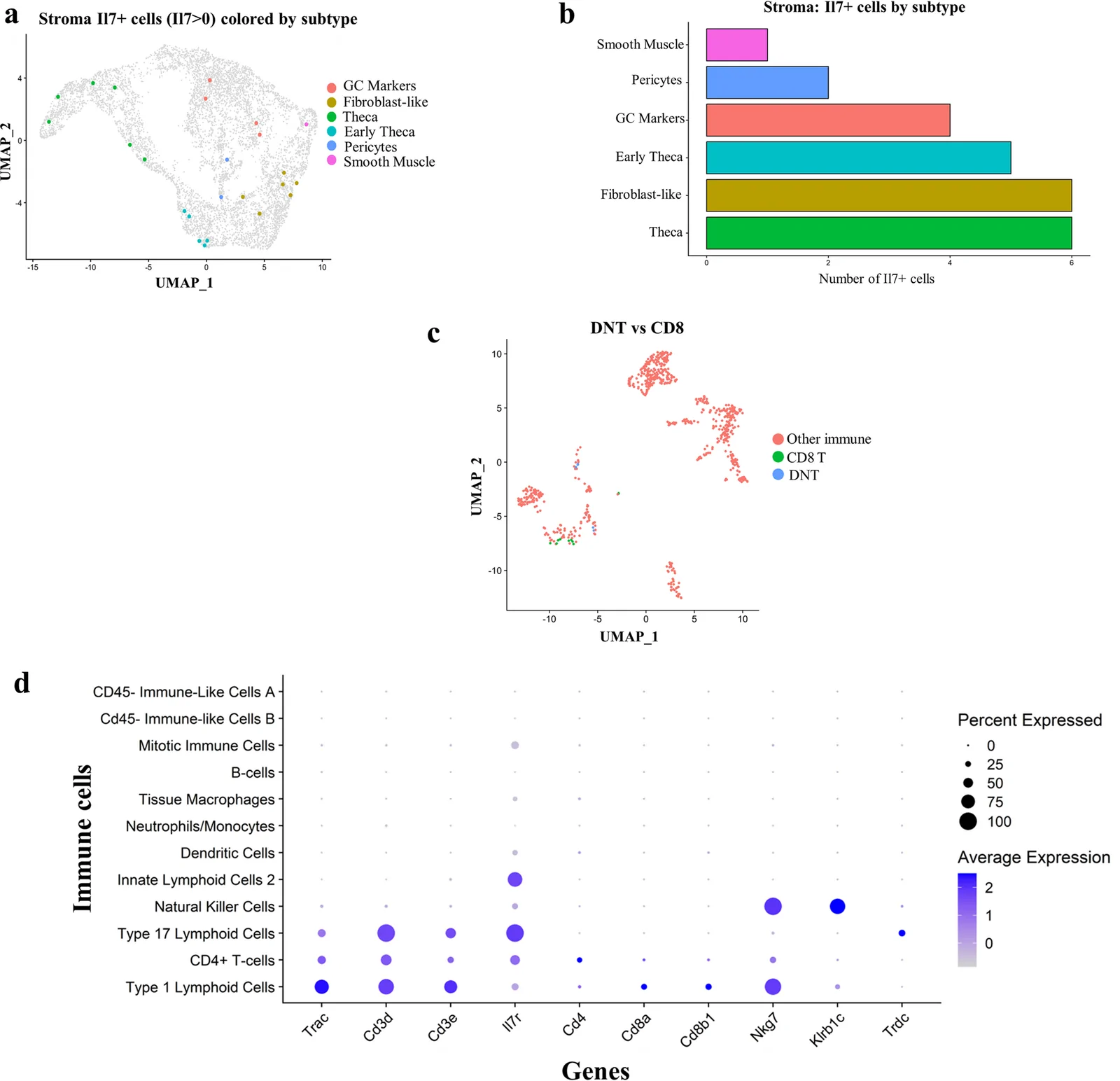
Re-analysis of published ovarian scRNA-seq identifies sparse Il7-expressing stromal subsets and an Il7r-enriched transcript-defined DNT-associated lymphoid population. **a** UMAP of ovarian stromal cells from the published GSE232309 dataset, analyzed here using ovaries from young mice (*n* = 4), with *Il7*-expressing cells (*Il7* > 0) highlighted and colored by stromal subtype (smooth muscle (magenta), pericytes (blue), GC markers (salmon), early theca (teal), fibroblast-like (olive-gold), theca (green)); all other stromal non-Il7-positive cells are shown in gray. Il7-positive cells were infrequent in this dataset and are presented as supportive. **b** Bar plot quantifying *Il7*⁺ stromal cell numbers per stromal subtype, using the color mapping from panel A. **c** UMAP of the ovarian immune compartment showing the distribution of CD4⁺ T cells, CD8⁺ T cells, and DNTs within the lymphoid region relative to other immune populations. **d** Dot plot of canonical T cell and cytotoxic lymphocyte markers across immune clusters, including *Il7r*, lineage markers (*Trac*, *Cd3d/e*, *Cd4*, *Cd8a/b1*), and cytotoxic/NK-associated markers (*Nkg7*, *Klrk1*). Dot size reflects the proportion of cells expressing each gene; color reflects scaled average expression

To define candidate ovarian microenvironmental signals underlying the CD8 expression program, we analyzed a published ovarian single-cell RNA-seq dataset (GSE232309 [25]), focusing on ovaries from young mice (*n* = 4) as supportive context with the analysis restricted to descriptive mapping and summary visualization of candidate IL-7-associated and lymphoid features. Within the stromal compartment, *Il7* transcripts were detectable but sparse, and distributed across multiple stromal subtypes. Because Il7-positive cells were infrequent in this dataset, we interpret these findings as supportive of candidate IL-7-associated microenvironmental features. *Il7*-positive stromal cells comprised 24 of 7,308 stromal cells overall (0.33%) in the re-analyzed dataset. These cells were distributed across several stromal subtypes, with the largest counts in theca, fibroblast-like, and early theca populations (Fig. 4a-b), consistent with sparse *Il7* expression across defined stromal subsets and compatible with local IL-7 availability in the ovarian microenvironment. We therefore interpret this data cautiously as supportive of possible local IL-7-associated conditioning cues.

Within the immune compartment, a transcript-based DNT population, defined by high *Trac* and *Cd3d/e* with low *Cd4* and *Cd8a/b1* and minimal NK-associated signal, was mapped within the lymphoid region of the immune UMAP (Fig. 4c). Marker-based annotation confirmed retention of core T-lineage features, with partial cytotoxic/NK-associated transcript co-expression in a subset (Fig. 4d, Fig. S6a). *Il7r* was enriched within this DNT-associated population relative to other lymphoid clusters (Fig. S6b), supporting that a transcript-defined DNT-associated lymphoid population in the ovary may be positioned to respond to local IL-7-associated cues.

Examination of the stromal compartment revealed that *Cxcl12*, *Cxcl16*, *Icam1*, *Vcam1*, *Cxcl10*, and *H2-Ab1* were enriched within fibroblast-like and theca-lineage subsets, with limited expression across other stromal populations (Fig. S7a, Fig. S8a, ). Ovarian lymphoid clusters co-expressed the corresponding receptors *Cxcr4*, *Cxcr6*, and *Itga4* alongside *Il7r* (Fig. S7b, Fig. S8b, ). Within granulosa subsets, *Cxcl12*, *Icam1*, and *Cxcl10* were detectable but restricted to specific subregions (Fig. S9a), and *Icam1*, *Vcam1*, and *Cxcl12* were present within endothelial regions (Fig. S8b), identifying vascular interfaces as additional candidate sites of leukocyte interaction. Projection of CD4^+^ T cells, CD8^+^ T cells, and DNTs onto the immune UMAP showed overlap within a shared lymphoid transcriptional neighborhood, with DNTs and CD8^+^ T cells occupying overlapping but non-identical positions (Fig. S9). These findings are consistent with transcriptomic proximity within the dataset. Together, these findings provide support for stromal, endothelial, and lymphoid transcriptomic features compatible with local chemokine-, adhesion-, and IL-7-associated conditioning cues in the ovary.

### IL-7R is required for CD8 upregulation in ovarian-homed donor DNTs and shapes endogenous ovarian DNT: CD8^**+**^ T cell balance

**Fig. 5:**
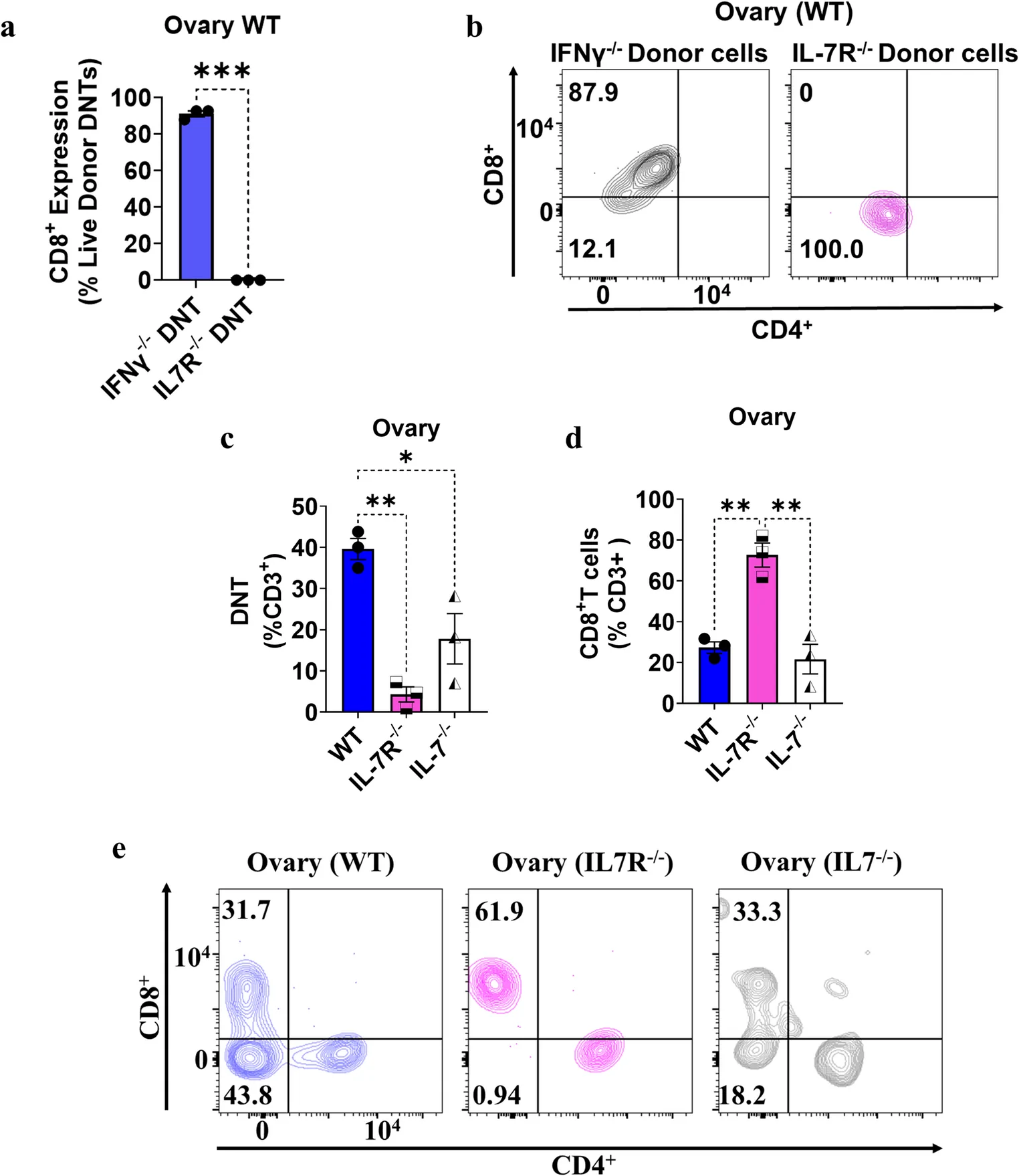
IL-7R supports efficient CD8 upregulation in ovarian-homed donor DNTs and its deficiency alters endogenous ovarian DNT: CD8^+^T cell balance.**a** Proportion of CD8-expressing donor cells in WT recipient ovaries following transfer of IFN-γ^−/−^ versus IL-7R^−/−^ DNTs (*n* = 3 mice; Mann-Whitney test). **b** Representative CD4 versus CD8 quadrant plots of donor IFN-γ^−/−^ (left) and IL-7R^−/−^ (right) cells in WT recipient ovaries; numbers indicate gate frequencies. **c**, **d** Endogenous ovarian DNT frequency (**c**) and CD8^+^ T cell frequency (**d**), expressed as a percentage of CD3^+^ T cells, in WT, IL-7R^−/−^, and IL-7^−/−^ mice (*n* = 3 mice per group; one-way ANOVA with Tukey post hoc). **e** Representative CD4 versus CD8 quadrant plots from WT, IL-7R^−/−^, and IL-7^−/−^ ovaries showing shifts in DNT and CD8^+^ T cell populations and anchored to FMO-defined thresholds; numbers indicate gate frequencies. Bars show mean ± SEM. *P < 0.05, **P < 0.01, ***P < 0.001

Given the *Il7r* enrichment on ovarian DNTs and scRNA-seq-based evidence for stromal IL-7 sources, we tested whether IL-7R signaling is functionally required for CD8 upregulation after ovarian localization. IL-7R^−/−^ donor DNTs transferred into WT recipients showed significantly reduced CD8 expression in the ovary relative to IFN-γ^−/−^ donors (Fig. 5a-b), establishing a DNT-intrinsic requirement for IL-7R in ovarian CD8 induction.

To assess the role of the IL-7 axis in steady-state ovarian T cell composition, ovaries from WT, IL-7R^−/−^, and IL-7^−/−^ mice were profiled in the absence of adoptive transfer. IL-7R deficiency was associated with a marked reduction in ovarian DNT frequency and a reciprocal increase in ovarian CD8⁺ T cell frequency relative to WT (Fig. 5c-e). IL-7^−/−^ ovaries also showed reduced DNT frequency, but without the marked increase in CD8⁺ T cell frequency observed in IL-7R^−/−^ mice (Fig. 5c-e), indicating that IL-7 and IL-7R deficiency impose distinct effects on ovarian T cell homeostasis. These findings support an IL-7R requirement for efficient ovarian CD8 induction and implicate the IL-7 axis in shaping the steady-state ovarian DNT: CD8⁺ T cell balance. These data do not by themselves establish that IL-7 is sufficient to drive this response, and they do not identify the ovarian cell population responsible for the relevant conditioning cue.

**Fig. 6:**
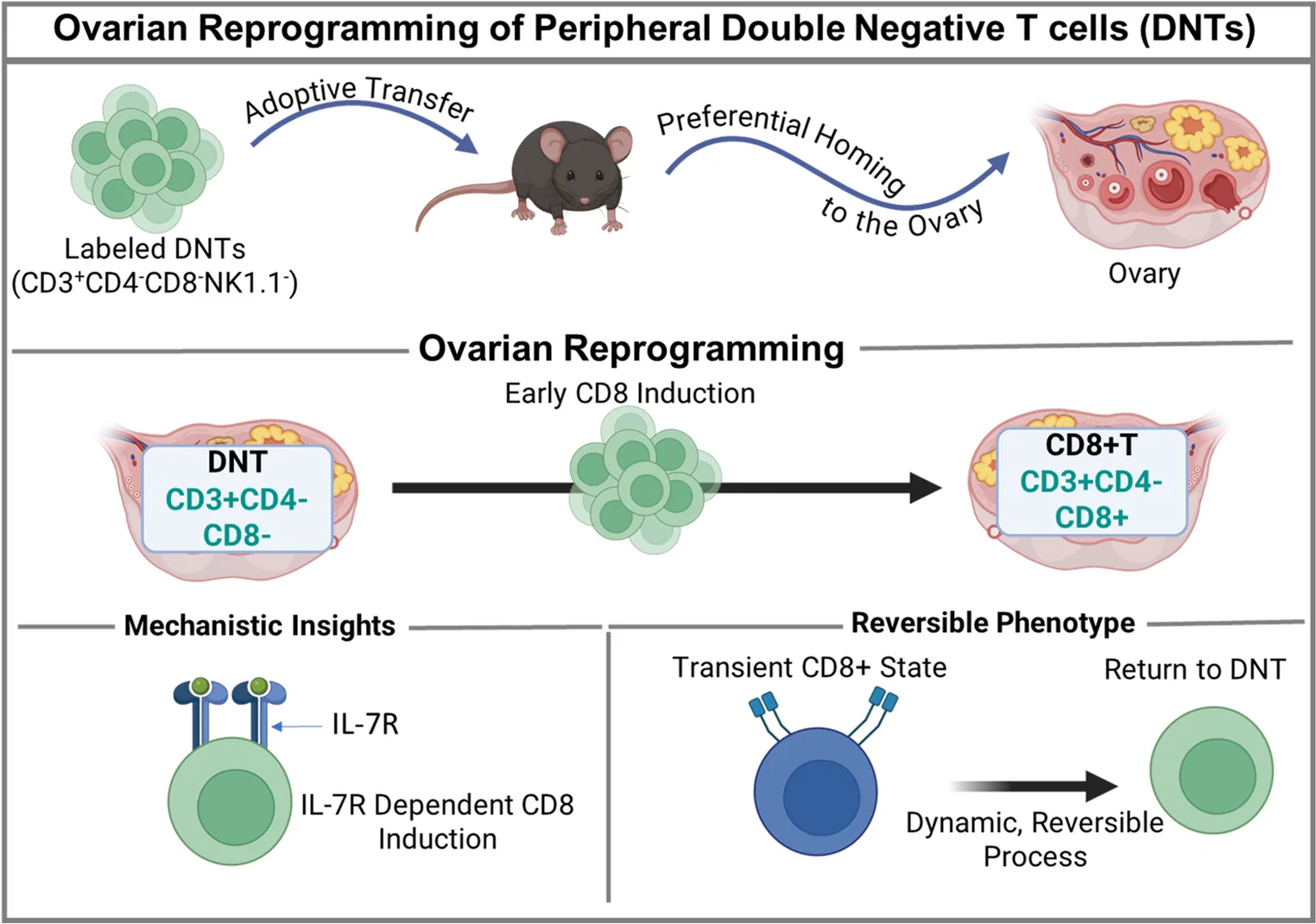
Ovarian localization drives IL-7R-dependent CD8 expression in peripheral DNTs. Schematic of the experimental design and main findings. FACS-sorted peripheral DNTs (CD3^+^CD4^−^CD8^−^NK1.1^⁻^) were labeled and transferred into recipient mice. Following transfer, donor DNTs accumulated preferentially in the ovary. After ovarian entry, a subset of donor cells expressed surface CD8, generating a donor-derived CD8-expressing population (CD3^+^CD4^−^CD8^+^) that resolved toward a CD4^−^CD8^−^ phenotype by day 14. IL-7R was required for this CD8 upregulation, suggesting IL-7R-dependent signaling as a functional requirement for ovarian CD8 expression on infiltrating peripheral DNTs

A summary of the experimental workflow and main findings is provided in Fig. 6.

## Discussion

This study supports that the ovarian microenvironment can condition the surface phenotype of peripheral T cells after tissue entry. Using adoptive transfer of peripheral DNTs, we found that these cells preferentially accumulate in the ovary relative to the uterus, spleen, and reproductive tissue-draining lymph nodes, and that ovarian localization is followed by de novo CD8 surface expression within four days. Importantly, CD8 expression declined by day 14, with donor cells returning toward a CD4^−^CD8^−^ phenotype, consistent with a dynamic, ovarian-conditioned change in surface coreceptor expression. These findings are supported by in vitro coculture data showing that dissociated ovarian cells are sufficient to promote CD8 upregulation on sorted DNTs in the absence of in vivo trafficking, indicating that the relevant signals are cell-associated and not dependent on systemic cues. Together, the data support a model in which ovarian-localized cues are associated with CD8 induction in infiltrating peripheral DNTs and are compatible with contributions from cytokine-, chemokine-, and adhesion-mediated conditioning signals. This is consistent with a growing body of work showing that non-lymphoid tissues can remodel infiltrating T cells through integrated microenvironmental signals distinct from those encountered in secondary lymphoid organs [11, 43].

The return to a CD4^−^CD8^−^ phenotype by day 14 indicates that CD8 surface expression on ovarian-homed donor DNTs is not a stable lineage commitment but a dynamic, context-dependent response to local cues [15, 44–46]. Because donor cells were analyzed at the endpoint, we cannot formally exclude contribution from a rare pre-existing donor subpopulation; however, the immediate pre-transfer donor phenotype and the ex vivo induction assay together favor interpretation as induced CD8 acquisition within a purified DNT donor population. This is also consistent with prior observations that CD8 surface levels can be modulated by cytokine signals in activated T cells, with expression declining once the driving stimulus is withdrawn [17, 19]. In the ovary, the cyclic nature of the tissue environment, which undergoes recurrent inflammatory-like remodeling across the reproductive cycle, is consistent with a conditioning signal that is itself periodic and non-sustained. Defining whether CD8 is lost because of reduced local cytokine availability, competing immune signals, or exit from the relevant microenvironment will require time-resolved donor tracking. The magnitude of the response also varied with recipient IFN-γ signaling status. Specifically, IFN-γ^−/−^ recipients showed the highest proportion of CD8-expressing donor cells and the strongest CD8α surface levels, while the persistent CD4^−^CD8^−^ fraction was largest in IFNγR^−/−^ recipients, consistent with IFN-γ pathway activity constraining the magnitude of this response [47, 48]. Together, these findings indicate that ovarian CD8 induction is not fixed, but is tuned by the surrounding immune signaling environment. The stronger response in IFN-γ^−/−^ hosts also suggests that the modulation may be mediated through the donor cells. Further dissection of this modulation, including whether IFN-γ acts directly on donor DNTs or through changes in the ovarian stromal or immune environment, will be required to define the underlying mechanism.

Donor DNT-derived CD8^+^ cells in the ovary were phenotypically distinct from endogenous host ovarian CD8^+^ T cells across multiple surface markers, indicating that ovarian CD8 upregulation does not simply expand a host-like CD8^+^ phenotype. Donor-derived CD8^+^ cells showed a CD5^lo^ IL-7R^hi^ surface profile and a CD62L^hi^-biased differentiation distribution, in contrast to host ovarian CD8^+^ T cells, which were predominantly CD5^+^, showed minimal IL-7R expression, and were enriched for effector and effector-memory markers. The CD5^lo^ feature is consistent with reduced cumulative TCR signaling, suggesting that donor-derived cells represent an earlier, partially conditioned state and not a fully differentiated ovarian CD8^+^ population [49, 50]. Their higher IL-7R expression is also consistent with preserved responsiveness to homeostatic cytokine cues [15, 16]. In addition, the opposing correlation structure of CD8α, IL-7R, and CD5 in donor-derived versus host CD8⁺ T cells supports that these populations are regulated differently and represent distinct CD8-expressing states. The ovarian scRNA-seq provided supportive, but limited, evidence for candidate IL-7-associated conditioning cues. *Il7* transcripts were sparse and localized mainly to fibroblast-like and theca-lineage stromal subsets, consistent with localized stromal IL-7-associated signal availability described in lymphoid tissues [15, 16, 51]. *Il7r* was enriched in the DNT-associated lymphoid population relative to CD4^+^ and CD8^+^ T-cell clusters, and stromal and endothelial compartments also expressed chemokine and adhesion ligands, including Cxcl12, Cxcl16, Icam1, and Vcam1, with corresponding receptor expression in lymphoid cells [52–55]. Collectively, with the preferential ovarian accumulation of donor DNTs, these data support an ovarian microenvironment compatible with lymphocyte entry, retention, and cytokine responsiveness. However, because the dataset shows sparse Il7 signal and limited immune cell depth, these observations should be interpreted as hypothesis-generating and do not establish a spatially resolved niche or direct stromal-lymphoid interaction.

IL-7R was required for efficient CD8 upregulation after ovarian localization, as IL-7R^−/−^ donor cells showed markedly reduced CD8 expression in WT recipient ovaries compared with IFN-γ^−/−^ donors. This identifies a DNT-intrinsic signaling requirement and is consistent with the established role of the IL-7 axis in supporting T cell survival and responsiveness to tissue-conditioning cues [16]. In intact mice, IL-7R deficiency produced a marked reduction in ovarian DNT frequency alongside an increase in CD8^+^ T cell frequency, while IL-7^−/−^ mice showed a more modest shift without the same degree of CD8⁺ T cell enrichment. This difference suggests that IL-7Rα supports ovarian DNT homeostasis through signals beyond IL-7 alone. Though IL-7R is required for efficient CD8 upregulation, the present study does not rule out contributions from other ovarian cytokines or stromal signals. These findings also have broader relevance to transferred and engineered T cell therapies, where therapeutic efficacy depends not only on the transferred cell but also on how recipient tissues support cell persistence, phenotype, and function. Recent perspectives in CAR-T engineering, including mRNA-based CAR-T strategies [56], emphasize controllable receptor expression and improved safety, but also highlight the continuing need to understand how transferred T cells are maintained and functionally shaped after entering tissue microenvironments.

The regulatory capacity of DNTs in inflammatory and autoimmune contexts has been described across several tissue settings [8, 9, 57], and the present findings extend this to the ovary, supporting that ovarian localization promotes a phenotypically distinct, IL-7R-dependent requirement for CD8-expression in peripheral DNTs that is further shaped by the prevailing cytokine environment. Furthermore, recent large-scale single-cell profiling has further emphasized the heterogeneity of DNT populations across tissue and disease contexts, including a pan-cancer atlas identifying multiple transcriptionally distinct DNT states across human tumor microenvironments [58]. The observation that this phenotype shows a context-dependent response to local cues and is quantitatively tunable by immune signaling suggests that the ovarian DNT pool is not static but dynamically responsive to the local immune milieu. Disruption of this conditioning capacity, for example through chronic elevation of IFN-γ, loss of IL-7 availability, or altered stromal microenvironment architecture, could shift the ovarian DNT: CD8^+^ T cell balance in ways that compromise local immune homeostasis or contribute to reproductive dysfunction. This possibility is supported by prior work demonstrating that aberrant CD8^+^ T cell expansion in the setting of elevated IFN-γ is associated with reproductive pathology in mice [31], and suggests that the IL-7R-dependent DNT program described here may normally contribute to keep that balance in check. This literature emphasizes that DNT programs are highly context dependent across tissues and disease settings [59, 60], and we therefore interpret the ovarian phenotype as a distinct tissue-conditioning response. More broadly, our findings are supported by the concept that local microenvironments modulate immune cell behavior through integrated cytokine, chemokine, adhesion, stromal, and extracellular-matrix cues [61]. Though this principle is often discussed in tumor microenvironments, where immune cell function is regulated by coordinated signals from stromal, vascular, matrix, and immune compartments, similar tissue conditioning logic may also apply to non-malignant reproductive tissues undergoing cyclic remodeling. In the ovary, IL-7R-dependent CD8 induction may therefore represent one example of how local tissue cues can remodel infiltrating T cell states after entry.

This study has several limitations. IL-7 was identified at the transcript level in re-analyzed scRNA-seq data but was not validated at the protein level or mapped spatially in ovarian tissue, so the atlas should be interpreted as supportive not definitive. The donor IL-7R transfer experiment establishes a requirement for IL-7R but does not distinguish direct signaling from survival effects, and the ex vivo coculture assay cannot identify the responsible ovarian cell type or mediator. In addition, CD8 acquisition does not by itself establish stable lineage conversion, and time-resolved transcriptional or lineage-tracing approaches will be needed to define the trajectory and functional significance of this state. Also, whether the ovarian conditioning program described here is specific to DNTs or extends to other peripheral T-cell populations remains to be determined. A limitation of the adoptive transfer design is that donor DNTs were administered intraperitoneally. Because the ovary is anatomically adjacent to the peritoneal compartment, proximity to the delivery route may contribute to donor-cell recovery observed in the ovary. Therefore, we interpret these data as preferential ovarian recovery after i.p. transfer. Furthermore, since day 14 analysis focused on ovarian donor cells, these data support phenotype resolution within the ovarian donor compartment but do not define later donor cell persistence across tissues. Nevertheless, the identification of an IL-7R requirement for CD8 upregulation as a feature of ovarian T cell conditioning provides a defined mechanistic entry point and framework for addressing these questions and understanding how the ovarian microenvironment shapes infiltrating T cell phenotypes under homeostatic conditions.

This study supports that the ovary can act as an active immune-conditioning site in which tissue entry is associated with CD8 upregulation in peripheral DNTs, with IL-7R signaling contributing to efficient induction in this setting. IL-7R deficiency reduces CD8 upregulation in donor DNTs and alters the endogenous ovarian DNT: CD8^+^ T cell balance, identifying an IL-7R-dependent signaling requirement for ovarian T cell phenotype. These findings provide a framework for interpreting ovarian T cell phenotypes in homeostasis, raise testable questions about how ovarian immune conditioning is regulated across the reproductive cycle, and identify candidate mechanisms through which disruption of the ovarian microenvironment could contribute to T cell imbalance in reproductive pathology.

## Supplementary Information

Below is the link to the electronic supplementary material.

Supplementary Material 1.

## Data Availability

The datasets generated during and/or analyzed during the current study are not publicly available as the primary flow cytometric data files are held in laboratory experimental records that have not been submitted to a public repository, but are available from the corresponding author on reasonable request.

## Abbreviations

ACK: Ammonium-chloride-potassium (lysis buffer)
ANOVA: Analysis of variance
ARE: AU-rich element
CD: Cluster of differentiation
dLN: Draining lymph node
DNT: Double-negative T cell
EDTA: Ethylenediaminetetraacetic acid
FACS: Fluorescence-activated cell sorting
FBS: Fetal bovine serum
FMO: Fluorescence minus one
FSC: Forward scatter
GEO: Gene Expression Omnibus
GFP: Green fluorescent protein
IFN-γ: Interferon-gamma
IL-7: Interleukin-7
IL-7R: Interleukin-7 receptor
MFI: Mean fluorescence intensity
MHC: Major histocompatibility complex
NK: Natural killer
PBS: Phosphate-buffered saline
PMT: Photomultiplier tube
QT: Qtracker 525
RPMI: Roswell Park Memorial Institute (medium)
scRNA-seq: Single-cell RNA sequencing
SEM: Standard error of the mean
SSC: Side scatter
TCR: T cell receptor
UMAP: Uniform manifold approximation and projection
UTR: Untranslated region
WT: Wild-type
