# Supplementary material for: Ovarian localization drives IL-7R-dependent CD8 coreceptor expression in peripheral double-negative T cells
Source: Cell Mol Life Sci. 2026 Jun 16;83(1):315. doi: 10.1007/s00018-026-06295-x (PMC13507013; doi:10.1007/s00018-026-06295-x)
Supplement: Supplementary file 1 [file 18_2026_6295_MOESM1_ESM.pdf]

Supplementary Data

Ovarian localization drives IL-7R-dependent CD8 coreceptor expression in peripheral double-negative T cells

Supplementary Method

Table S1. Antibodies used for flow cytometry and cell sorting

Antibody	Fluorophore	Clone	Company
**CD45**	Alexa Fluor-700	30-F11	BioLegend
**CD3**	PE/dazzle594	17A2	BioLegend
**CD4**	APC-eFluor780	RM4-5	Invitrogen, ThermoFisher Scientific
**CD8a**	FITC	53-6.7	BioLegend
**CD8a**	APC	53-6.7	BioLegend
**CD8a**	PE-Cy7	53-6.7	BioLegend
**NK1.1**	Brilliant Violet 711	PK136	BioLegend
**CD5**	BV421	53-7.3	BioLegend
**CD44**	BUV395	IM7	BD Biosciences
**CD62L**	PerCPCy5.5	MEL-14	BioLegend
**CD62L**	PE-Cy5	MEL-14	BioLegend
**CD127**	BUV737	SB/199	BD Biosciences
**Zombie**	Aqua	-	BioLegend

Supplementary Results

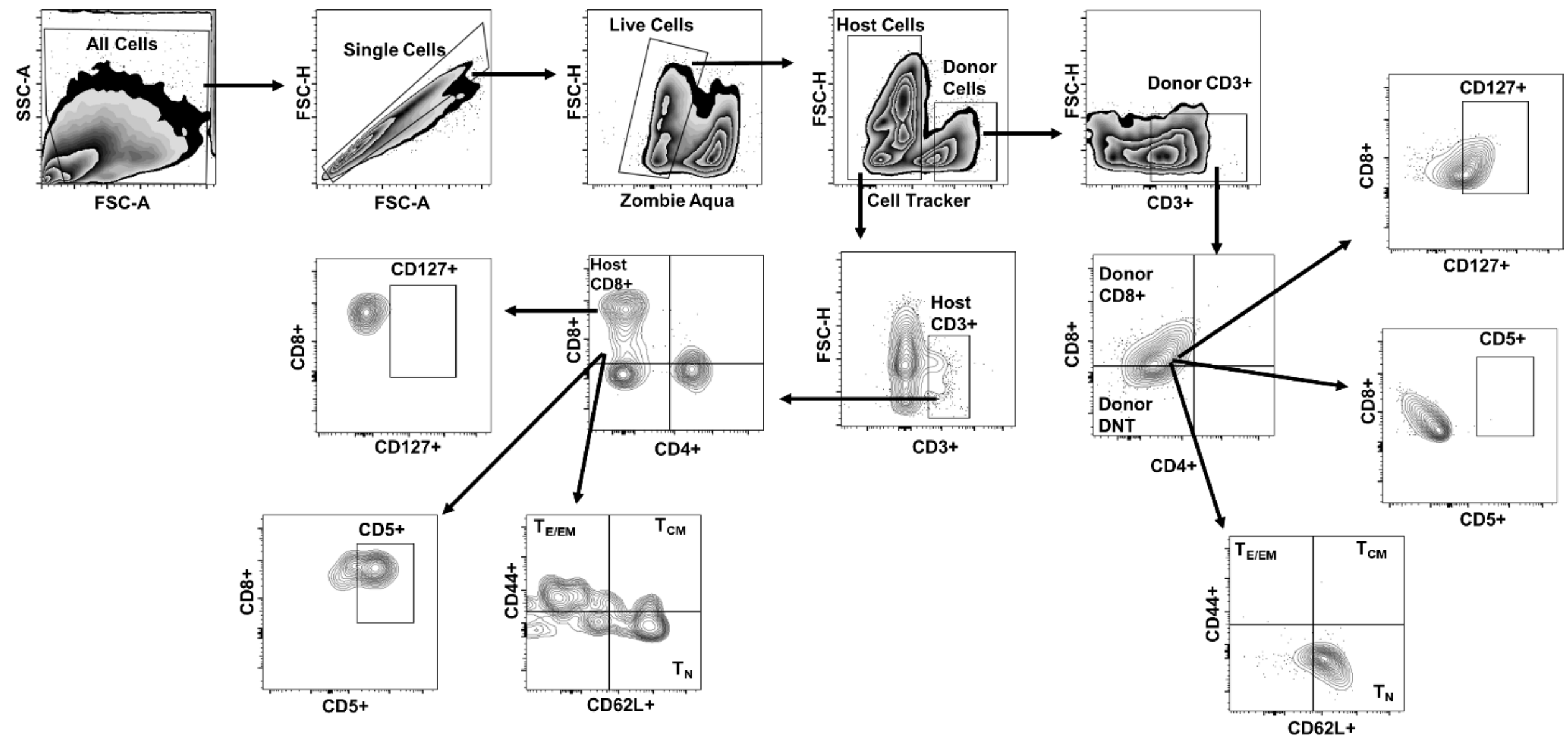

Figure S1. **Representative gating strategy for adoptive-transfer donor tracking and downstream phenotypic analysis.** Single cell suspensions from recipient ovaries were gated sequentially on all cells, single cells, and live cells, followed by separation of host and donor populations based on donor label. Donor cells were then gated as $CD3^+$ events and further resolved into donor $CD8^+$ and donor DNT subsets by CD4 versus CD8 expression. In parallel, host cells were gated to identify the host $CD8^+$ compartment. Downstream phenotypic analysis of donor- and host-derived $CD8^+$ T cells included CD127/IL-7R, CD5, and differentiation-state gating using CD44 and CD62L to define effector/effector-memory ($T_{E/EM}$), central memory (T_{CM}), and naive (T_N) subsets. The ovary is shown as the representative tissue, and the same donor tracking hierarchy was applied across tissues in adoptive transfer experiments.

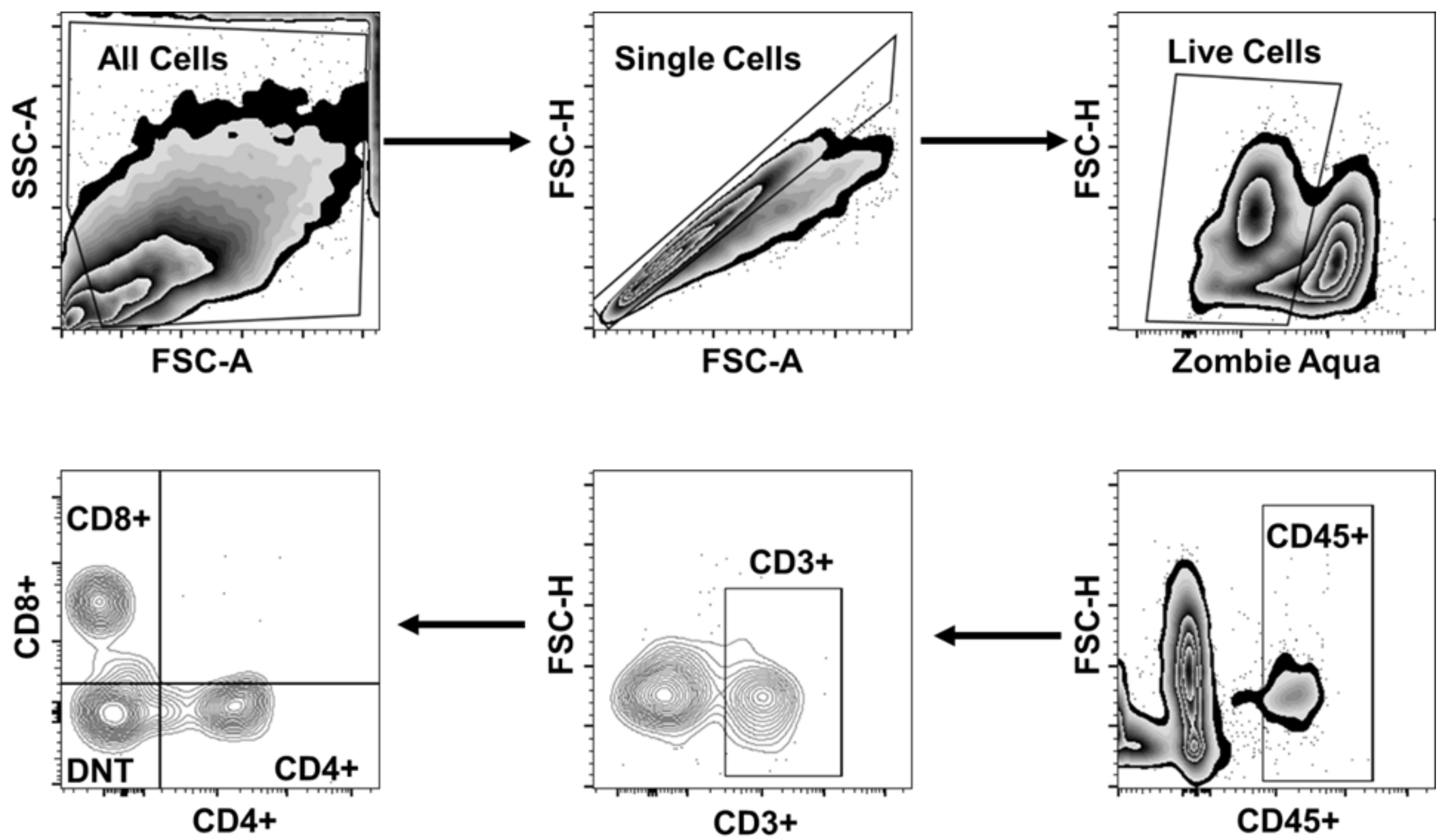

Figure S2. **Representative gating strategy for endogenous ovarian T-cell subset analysis.** Single cell suspensions from ovaries were gated sequentially on all cells, single cells, and live cells, followed by selection of $CD45^+$ leukocytes and $CD3^+$ T cells. Within the ovarian $CD3^+$ compartment, cells were resolved by CD4 versus CD8 expression to identify $CD4^+$, $CD8^+$, and double-negative T cells (DNTs; $CD4^-CD8^-$). Representative downstream gates used for phenotypic analysis of endogenous ovarian $CD8^+$ T cells, including CD127/IL-7R expression, are shown. This gating strategy was used for the endogenous ovarian analyses in Fig. 5.

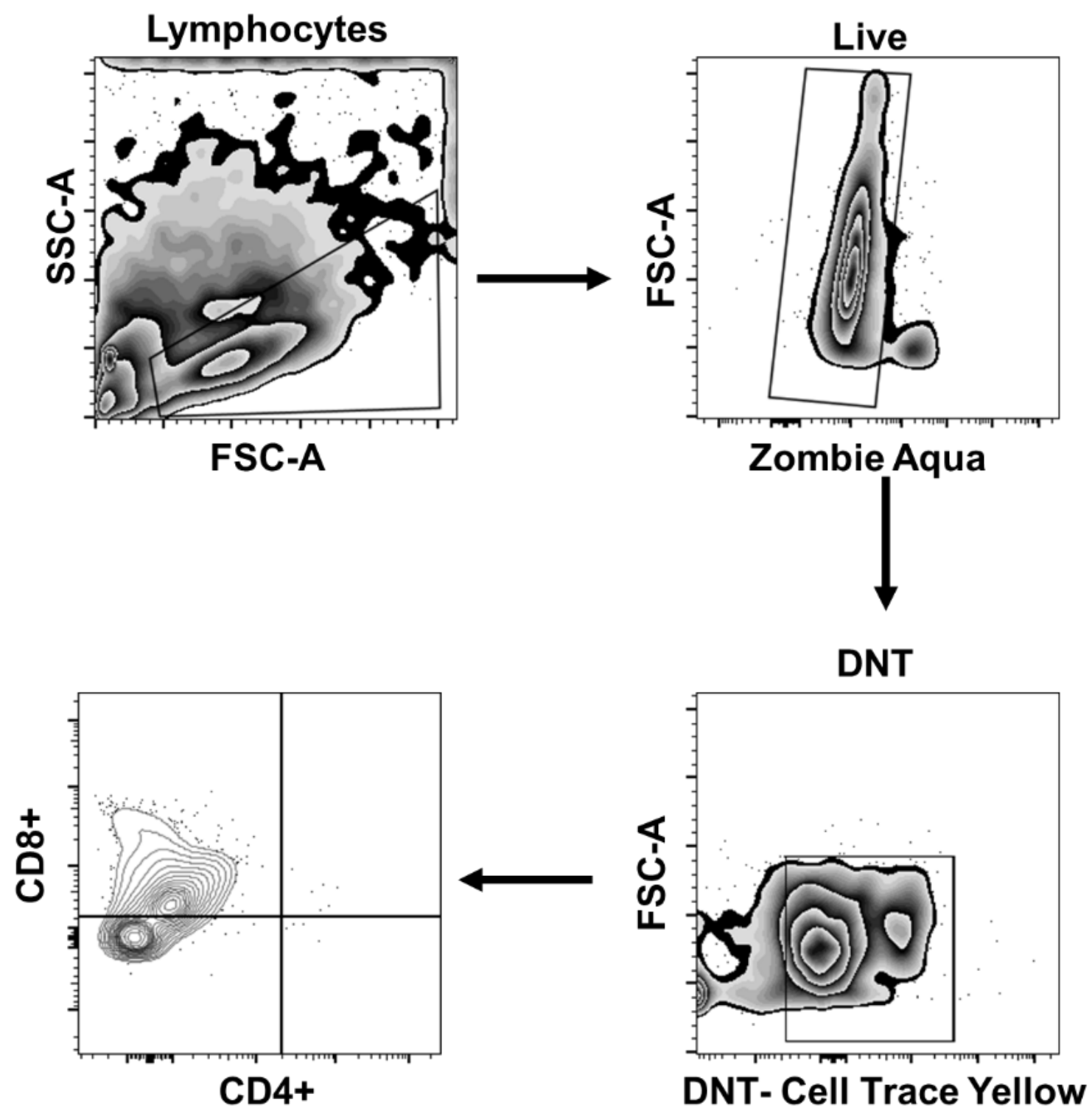

Figure S3. **Representative gating strategy for *ex vivo* ovarian coculture analysis.** Cells recovered from the 72 h coculture were gated sequentially on all cells, single cells, and live cells. Labeled donor cells were then identified by CellTrace Yellow positivity, followed by gating on $CD3^+$ donor T cells. Within the donor $CD3^+$ compartment, cells were resolved by CD4 versus CD8 expression to identify $CD8^+$ and $CD4^-CD8^-$ (DNT) populations after coculture. This gating strategy was used for the *ex vivo* coculture assay shown in Fig. S1.

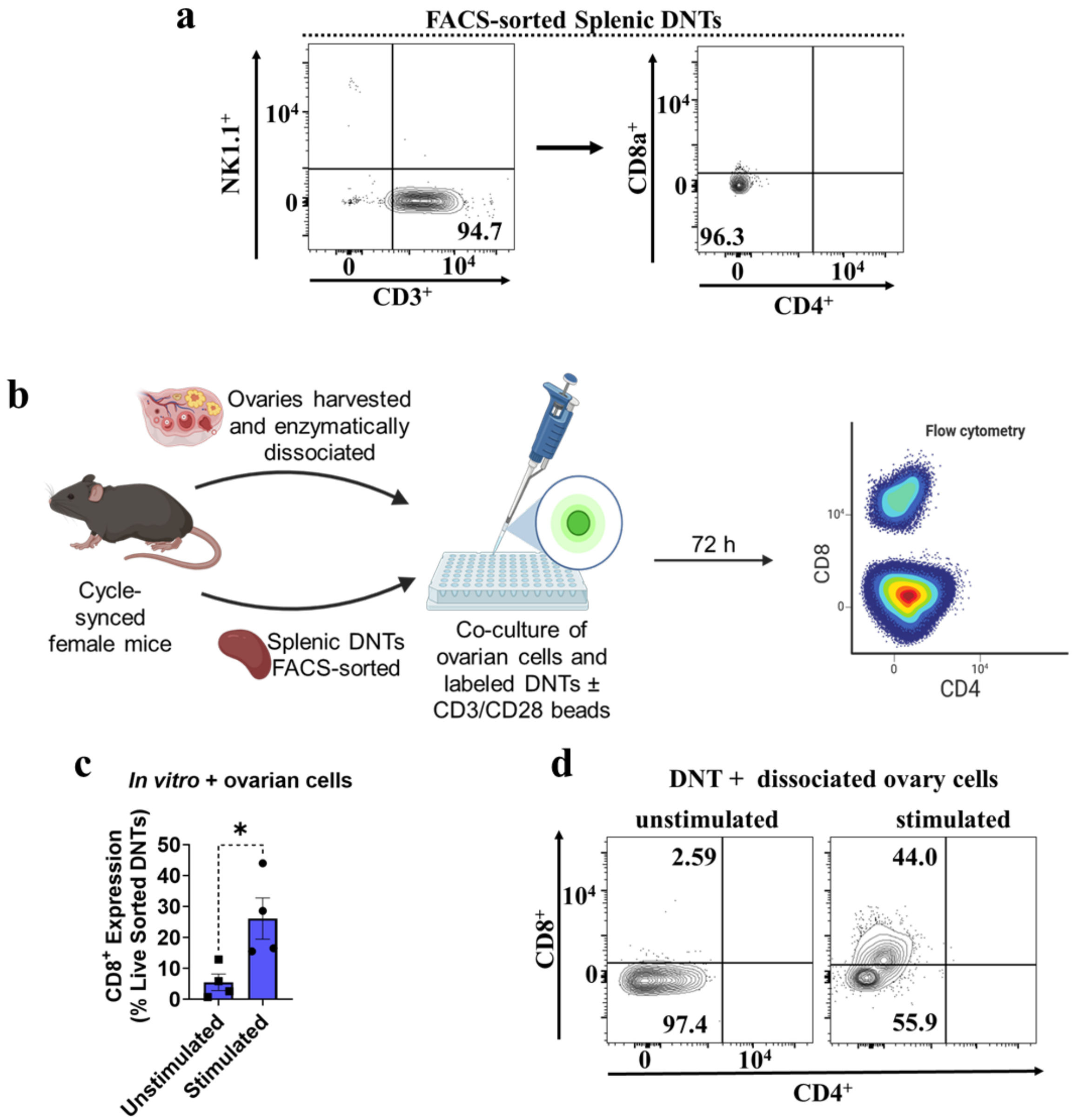

Figure S4 Post-sort purity of FACS-sorted splenic DNTs and ex vivo ovarian coculture sufficiency assay. (a) Representative post-sort purity plots for FACS-sorted splenic DNTs immediately prior to adoptive transfer or culture. Sorted immediate pre-injection donor cells are $CD3^{+}NK1.1^{-}$ and, within this gate, are $CD4^{-}CD8\alpha^{-}$, confirming exclusion of $NK1.1^{+}$ cells and a double-negative phenotype at baseline. **(b)** Schematic showing isolation of cells and tissues and coculture of labeled DNTs with dissociated ovarian cells for 72 h. **(c)** Quantification of CD8 expression on live, sorted DNTs following *ex vivo* coculture with dissociated ovarian cells under unstimulated conditions or with CD3/CD28 costimulation, reported as the percent $CD8^{+}$ among live sorted DNTs (n= 4 technical replicates). **(d)** Representative CD4 versus CD8 plots from the same co-cultures showing the distribution of DNTs that remain $CD4^{-}CD8^{-}$ versus those that induce CD8 with or without stimulation. Numbers indicate the percentage of events within the indicated gates. These *ex vivo* data are presented as a sufficiency assay and do not identify the specific ovarian cell type or mediator responsible for CD8 induction.

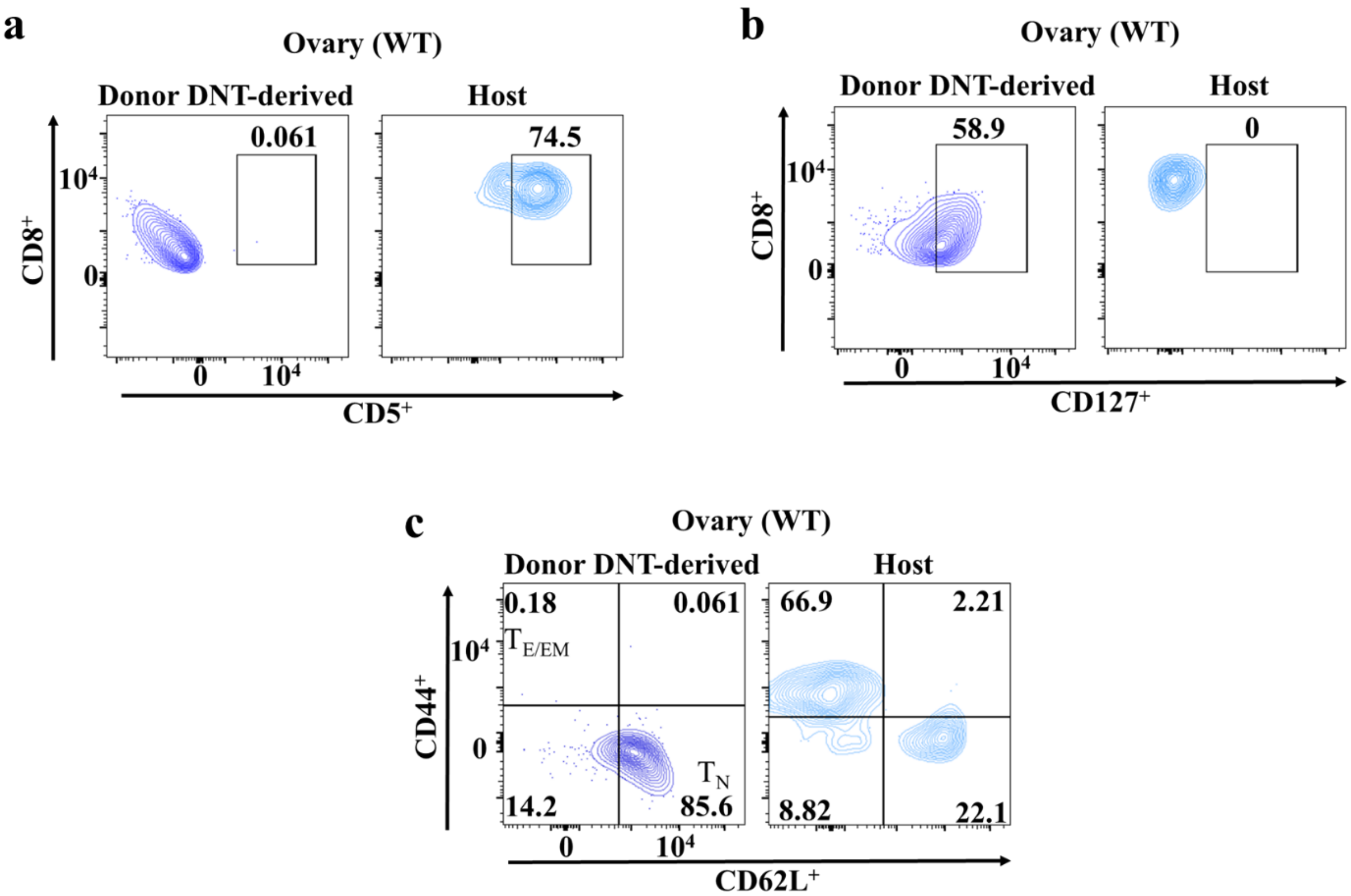

Figure S5 Representative gating plots for activation-associated markers and subset distribution in ovarian donor DNT-derived versus host CD8^{+} T cells (WT recipients, day 4). (**a-b**) Representative flow cytometry plots from WT mouse ovaries at day 4 post-transfer showing CD8^{+} cells expressing CD5 (**a**) and CD127/IL-7R (**b**) for donor DNT-derived CD8^{+} cells (left) and endogenous host ovarian CD8^{+} T cells (right). (**c**) Representative CD44 versus CD62L plots showing effector/effector-memory ($T_{E/EM}$; CD44^{hi}CD62L^{lo}) and naive (T_{N}; CD44^{lo}CD62L^{hi}) distributions within donor DNT-derived and host ovarian CD8^{+} T-cell compartments. Numbers indicate the percentage of events within the indicated gates. These comparisons define phenotypic divergence between donor-derived and host ovarian CD8-expressing cells and do not by themselves imply stable lineage conversion

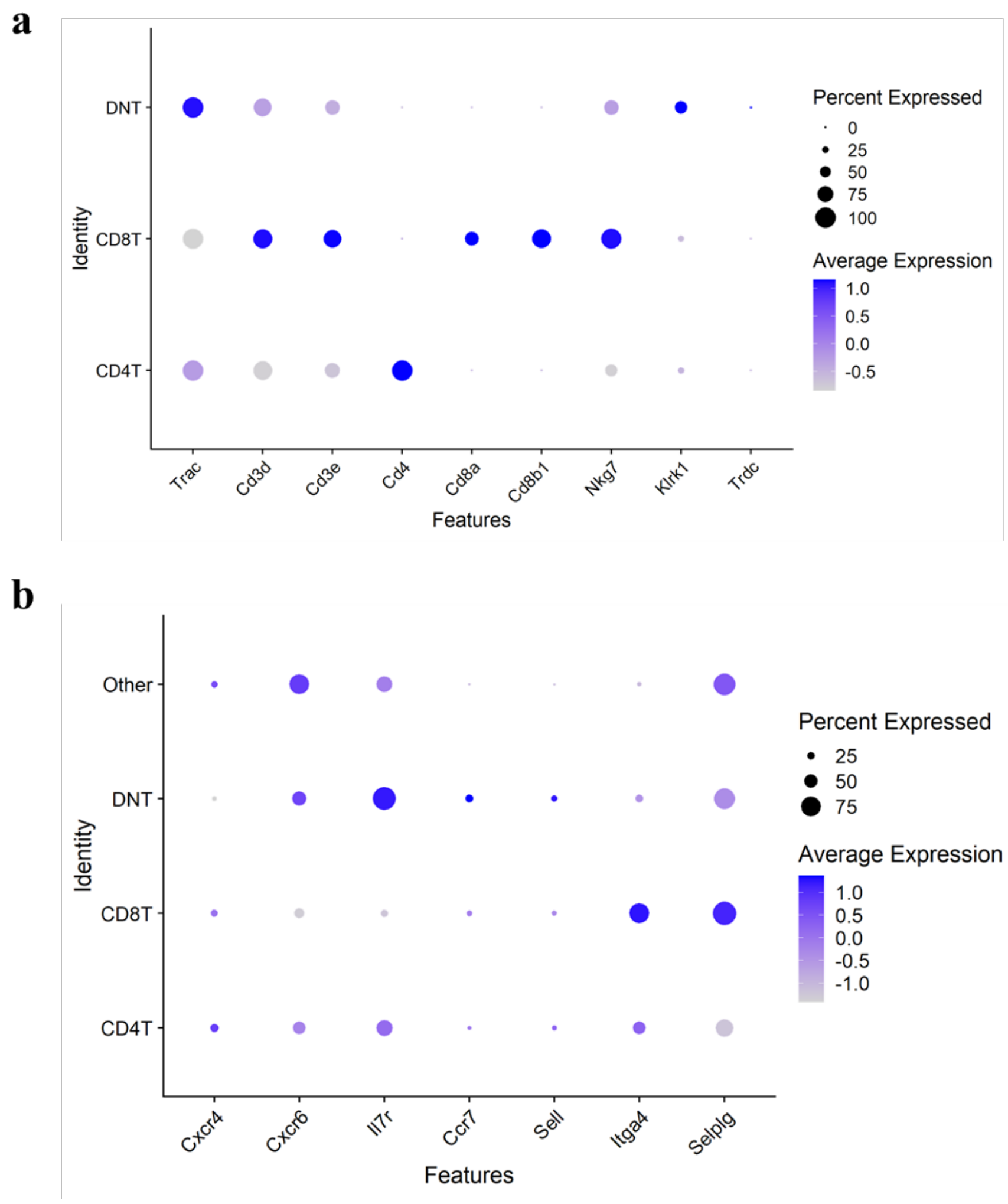

Figure S6 scRNA-seq dataset (GSE232309) identifies ovarian lymphoid clusters and receptor genes for ovarian DNTs (a) Dot plot of lineage and cytotoxic/NK-associated markers supporting marker-based annotation of DNTs relative to $CD4^{+}$ T and $CD8^{+}$ T cells. (**b**) Dot plot comparing *Il7r* and chemokine receptor/adhesion-associated genes across $CD4^{+}$ T cells, $CD8^{+}$ T cells, and DNTs. Dot size indicates percent expressing; color indicates scaled average expression.

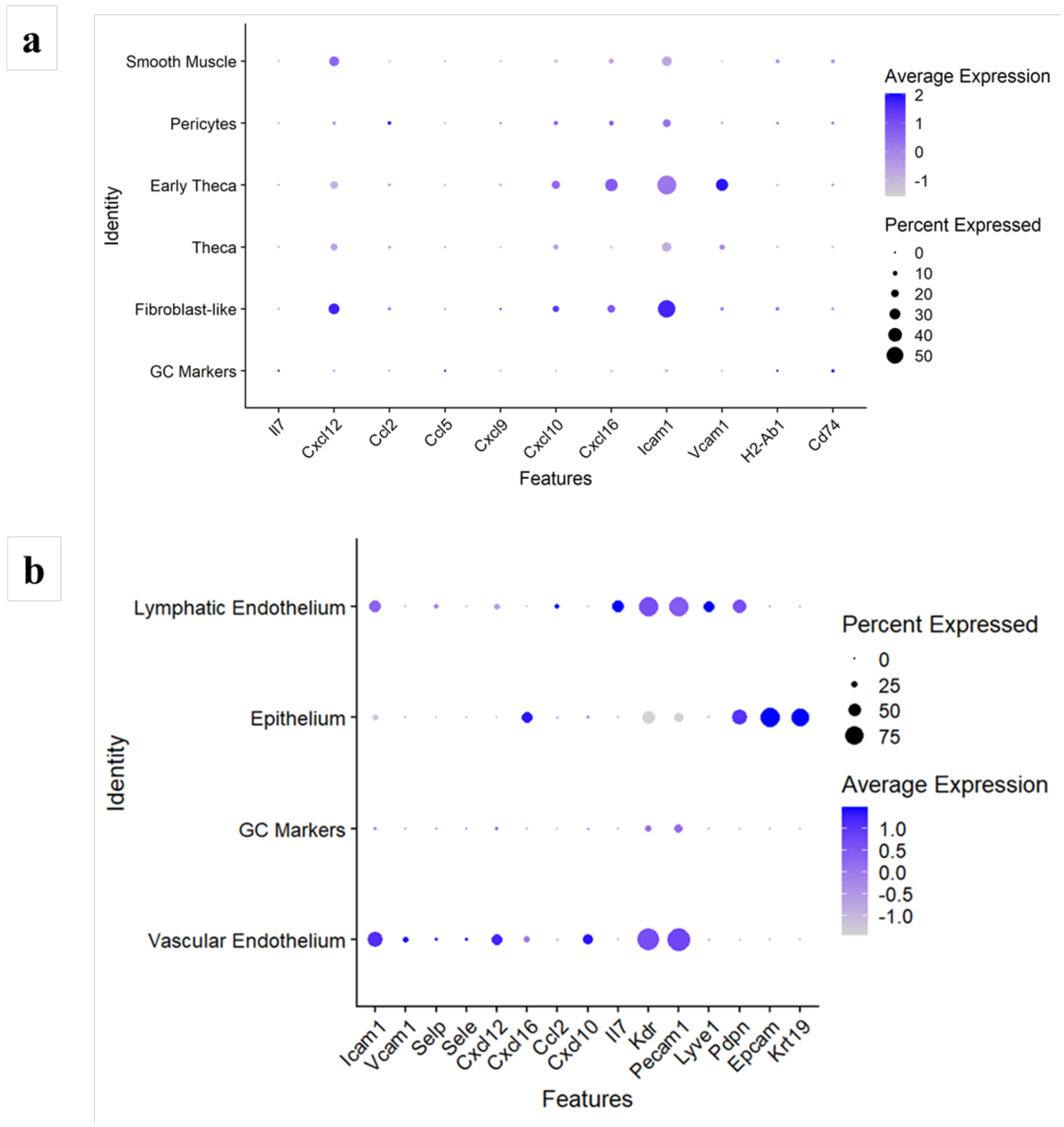

Figure S7. Stromal and endothelial chemokine/adhesion programs in the ovary (scRNA-seq, GSE232309). (a) Dot plot showing expression of chemokines, adhesion molecules, and immune-related genes across stromal subtypes (smooth muscle, pericytes, fibroblast-like, early theca, theca, GC marker-high). (**b**) Dot plot showing expression of vascular interaction genes across vascular endothelium, lymphatic endothelium, epithelium, and GC marker-high populations, including adhesion molecules (*Icam1*, *Vcam1*, *Sele/Selp*), chemokines (*Cxcl12*, *Cxcl16*), and identity markers (*Kdr*, *Pecam1* for endothelium; *Lyve1*, *Pdpn* for lymphatic endothelium; *Epcam*, *Krt19* for epithelium). Dot size indicates percent expressing; color indicates scaled average expression.

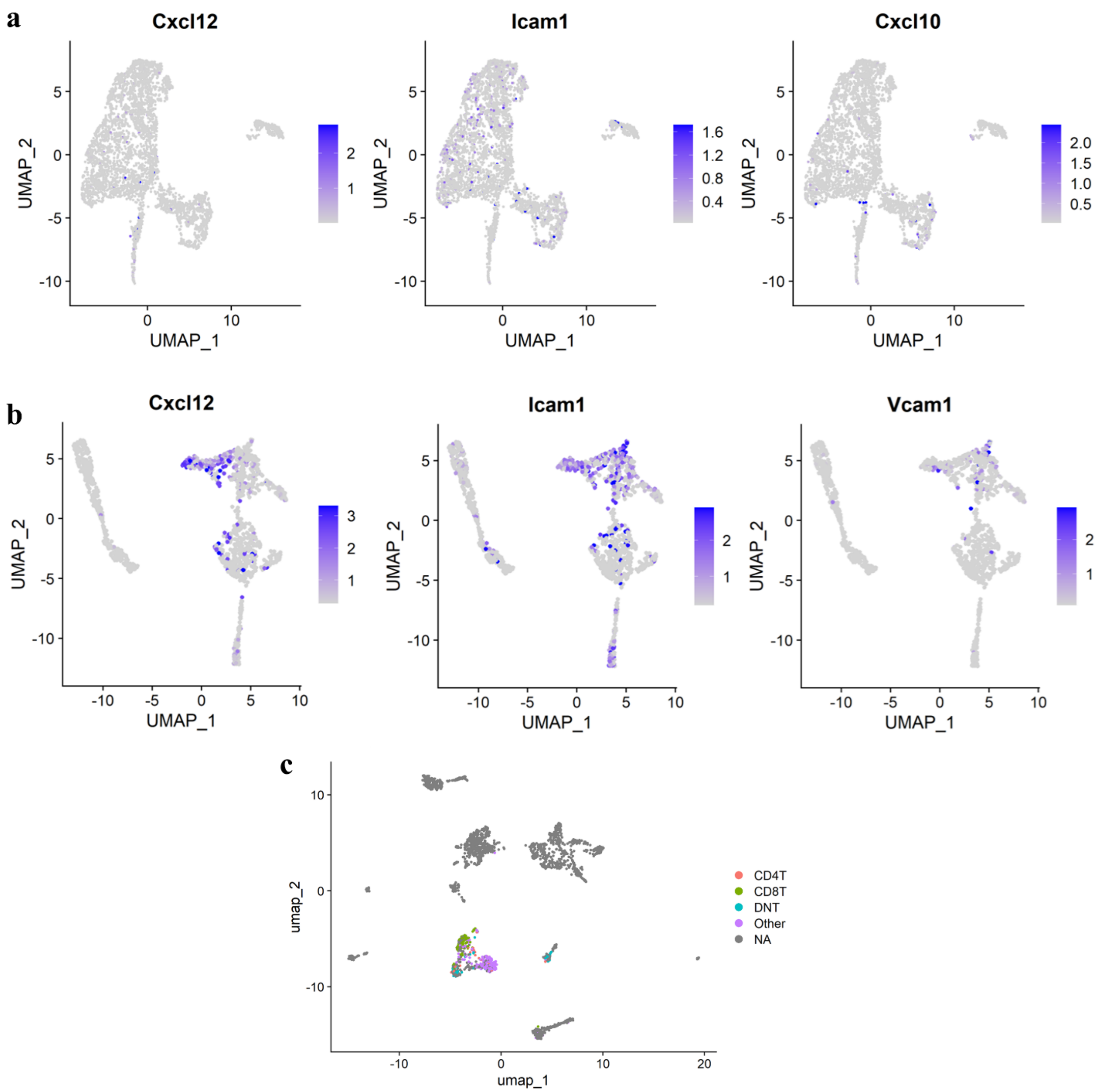

Figure S8. Ovarian stromal subsets express chemokine and adhesion cues alongside lymphoid cells enriched for the corresponding receptors. (**a**) UMAP plots from the ovarian stromal compartment showing expressions of *Cxcl12*, *Cxcl16*, *Icam1*, and *H2-Ab1*. Gray indicates low or undetectable expression; increasing blue intensity indicates higher normalized expression. Signal is enriched within defined stromal subsets, indicating non-uniform transcript enrichment across stromal states. (**b**) UMAP plots of the ovarian lymphoid compartment showing expression of *Cxcr4*, *Cxcr6*, *Il7r*, and *Itga4*, enriched within lymphoid clusters, consistent with co-expression of chemokine receptors, adhesion-associated receptors, and IL-7R in ovarian T cells.

a

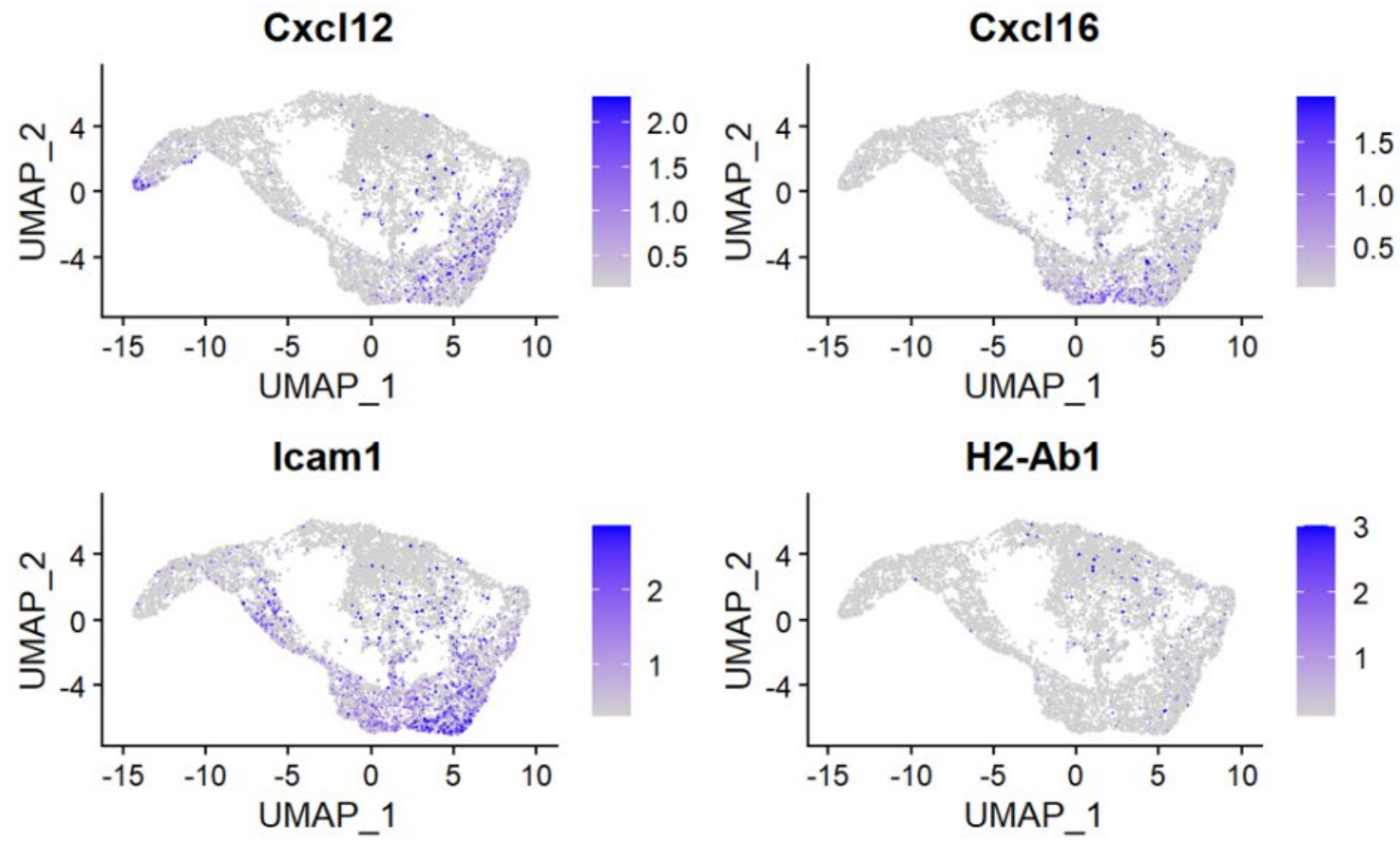

b

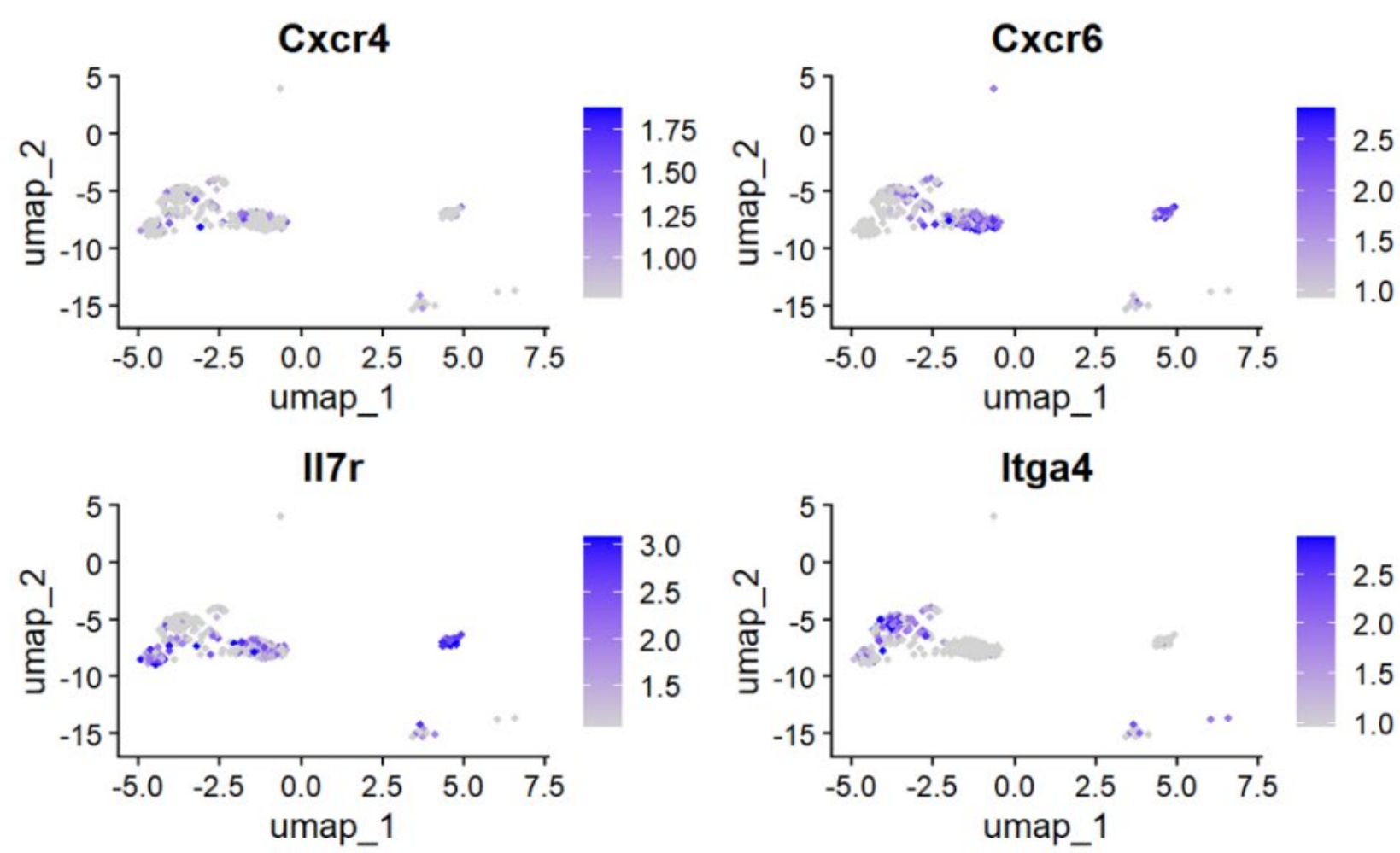

Figure S9. Granulosa and endothelial subregions express chemokine and adhesion signatures, and DNTs and CD8⁺ T cells occupy partially overlapping positions within the immune UMAP. (**a**) UMAP plots showing *Cxcl12*, *Icam1*, and *Cxcl10* expression across granulosa cell subsets; signal is concentrated in specific subregions. Gray indicates low or undetectable expression; increasing blue intensity indicates higher normalized expression. (**b**) UMAP plots from the endothelial compartment showing *Icam1*, *Vcam1*, and *Cxcl12* expression, consistent with vascular-associated leukocyte interaction cues. (**c**) UMAP of ovarian immune cells annotated by T cell class, showing $CD4^+$ T cells, $CD8^+$ T cells, and DNTs occupy partially overlapping positions within a discrete lymphoid region; other indicates immune populations outside these T cell classes; gray points indicate unassigned cells.
